# Analysis of structure and conservation for supporting functional evaluation of PMS2 missense variants

**DOI:** 10.1038/s41431-026-02072-3

**Published:** 2026-03-18

**Authors:** Nicholas Zeuzem, Manon Quilan, Mev Dominguez-Valentin, Stéphanie Baert-Desurmont, Madleen Horlacher, Adriana Della Valle, Patricia Esperon, Florencia Neffa, Taisa Manuela Bonfim Machado-Lopes, Ivana Lucia de Oliveira Nascimento, Maria Betânia Pereira Toralles, Thaís Ferreira Bomfim-Palma, Walter Hernan Pavicic, Carlos A. Vaccaro, Florencia Spirandelli, Carlos Santamaria-Quesada, Geiner Jimenez, Felipe Vaca-Paniagua, Sandra Perdomo, Juan Javier López Rivera, Giovana Tardin Torrezan, Dirce Maria Carraro, Angela Brieger, Hubert Serve, Alexandra Martins, Guido Plotz

**Affiliations:** 1 Department of Internal Medicine 1, University Hospital, Goethe University, Frankfurt am Main, Germany; 2 Univ Rouen Normandie, Inserm U1245, FHU G4 Génomique, F-76000 Rouen, France; 3 Department of Tumor Biology, Institute of Cancer Research, The Norwegian Radium Hospital, 0379 Oslo, Norway; 4 Univ Rouen Normandie, Inserm U1245, CHU Rouen, Department of Genetics, F-76000 Rouen, France; 5 Centro de Oncogenetica Uruguayo. Hospital Central de las Fuerzas Armadas, Montevideo, Uruguay; 6 Molecular Genetic Unit, School of Chemistry, Universidad de la República, Montevideo, Uruguay; 7 Laboratório de Imunologia e Biologia Molecular, Federal University of Bahia, Salvador-, BA, Brazil; 8 Instituto de Medicina Traslacional e Ingeniería Biomédica (IMTIB), Hospital Italiano de Buenos Aires (HIBA), Instituto Universitario Hospital Italiano de Buenos Aires (IUHI), Consejo Nacional de Investigaciones Científicas y Técnicas (CONICET), Autonomous city, Ciudad Autónoma de Buenos Aires Argentina; 9 Consultorio Privado de Coloproctolgia, Hospital Español de Rosario, Rosario, Argentina; 10 Molecular Diagnostic Laboratory. National Children Hospital, San Jose, Costa Rica; 11 Hospital Dr. Rafael Angel Calderon Guardia, Caja Costarricense del Seguro Social, San Jose, Costa Rica; 12 Biomedicine Research Unit, Faculty of Superior Studies Iztacala, National Autonomous University of Mexico, Mexico City, 54090 Mexico; 13 National Laboratory of Health, Faculty of Superior Studies Iztacala, National Autonomous University of Mexico, Mexico City, 54090 Mexico; 14 Genomic Epidemiology Branch, International Agency for Research on Cancer (IARC/WHO), Lyon, France; 15 Medical Director of the Genetics Laboratory at Clinica Colsanitas, Clinica Universitaria Colombia, Keralty Group, Bogotá, Colombia; 16 Clinical and Functional Genomics Group, International Research Center, CIPE/A.C.Camargo Cancer Center, São Paulo, Brazil; 17 Department of Internal Medicine 2, University Hospital, Goethe University Frankfurt, Frankfurt am Main, Germany; 18 German Cancer Consortium (DKTK), Partner Site Frankfurt and German Cancer Research Center (DKFZ), Heidelberg, Germany; 19 Frankfurt Cancer Institute, Frankfurt, Germany

**Keywords:** Cancer genetics, Colon cancer, Cancer prevention, Predictive markers

## Abstract

Germline defects in mismatch repair (MMR) genes are known to significantly increase the risk of developing certain types of cancers, notably colorectal and endometrial cancers. These conditions are characterized under Lynch syndrome. Accurate diagnosis of this predisposition, along with meaningful predictive testing for family members, necessitates the identification of pathogenic variants. However, classifying small coding genetic variants identified in cancer patients is very challenging, specifically in the case of *PMS2* variants, since *PMS2* pathogenic variants display a lower penetrance and less severe phenotype and therefore a lower tumor burden in affected families. We have assembled clinical data on four *PMS2* missense variants of uncertain significance (VUS) identified in 23 patients (p.(Asp286Gly), p.(Asn335Ser), p.(Ile679Thr) and p.(Arg799Trp)). For these variants, functional testing was performed (RNA splicing, protein stability and catalytic activity). Since many protein ortholog sequences and accurate predictive models from AlphaFold2 are available, we also included a systematic analysis of residue conservation and structural role (ConStruct assessment). Overall, our findings indicate that p.(Asp286Gly) and p.(Arg799Trp) behave similarly to wild-type PMS2 and are thus probably neutral. In contrast, p.(Asn335Ser) and p.(Ile679Thr) conferred defects in protein expression or MMR activity. These could be explained by the relevant roles of these amino acids in MLH1-PMS2-N-terminal dimerization (p.Asn335) and C-terminal dimerization (p.Ile679). Our data thus suggest that p.(Asp286Gly) and p.(Arg799Trp) are benign, while the tumor risk in the other two variants remains to be established. Taken together, we suggest roadmaps for the individualized evaluation of difficult uncertain variants by comprising information from all available sources.

## Introduction

Lynch syndrome (MIM #120435) is a heritable condition associated with increased risk of different forms of cancer [1]. It results from a germline inactivating variant causing loss of function of one of four major DNA mismatch repair (MMR) genes: *MLH1*, *MSH2*, *MSH6*, and *PMS2* [2, 3**]**. Somatic loss of the wild-type allele causes cellular MMR deficiency and microsatellite instability (MSI) [4].

Although Lynch syndrome confers a strongly elevated cancer risk for the individual, it is difficult to diagnose based on clinical phenotype. This is particularly the case for carriers of PMS2 pathogenic variants (also called *path_PMS2*), because they have lower penetrance, milder phenotypes, later onset, and reduced familial burden compared to *path_MLH1* and *path_MSH2* carriers. Moreover, *path_PMS2* carriers may have a different tumor spectrum, with elevated prevalence of extra colonic localizations such as breast cancer and prostate cancer [5–9], although other studies have not found increased cancer rates [1, 10]. Notably, despite lower penetrance, *PMS2* defects still can cause early-onset cancer [11], and path_PMS2_carriers should therefore be identified for being offered appropriate surveillance measures [12].

The differences in phenotype have been attributed to a potential partial compensation of PMS2 defects by MLH3, which shows some degree of overlap in its biological activity with PMS2 [13, 14]. However, biochemical evidence for such compensation has not been found in recent investigations, confirming that MMR is indeed compromised in PMS2-deficient cells [15].

Lynch syndrome diagnosis relies on the identification of a germline pathogenic or likely pathogenic variant causing loss of function. Classification of variants follows a 5-tiered system [16]. However, a significant proportion of alterations identified in patients remain variants of uncertain significance (VUS) [3, 16].

For assessing the effects of coding variants, assays based on ectopically expressed human proteins are often used due to their direct applicability to human diseases [17–25]. Some of them have been validated concerning reproducibility [26, 27], and/or allow deduction of odds of pathogenicity that can be used for integrating the result by Bayesian statistics [27, 28] into a prior probability of pathogenicity based on in silico evaluations. Another approach uses reference variants [19, 20, 29].

In this work, we tested four *PMS2* missense variants characterized as VUS and identified in 23 individuals from Latin America and Europe. Although clinical information was available, it did not provide sufficient evidence. We therefore assessed RNA splicing, protein expression and functionality. We discuss these results in context with the available clinical information that has been gathered from the affected patients and their families, and intensively assessed the structural role of the altered residues.

## Materials and methods

### Cancer families

Unpublished data from hereditary cancer registries and published data from patients with suspected Lynch syndrome have been included in this work. The data include results from germline DNA testing, tumor testing (based on microsatellite instability analysis and/or immunohistochemistry) and family history [30–34].

Families that fulfilled the Amsterdam criteria and/or the Bethesda guidelines [35–37] were selected from 7 hereditary cancer registries: Hospital Italiano (Buenos Aires, Argentina), Oncológica Sanatorio Parque GO (Rosario, Argentina), Hospital de las Fuerzas Armadas (Montevideo, Uruguay), Laboratório de Imunologia e Biologia Molecular do Instituto de Ciências da Saúde da Universidade Federal (LABIMUNO/UFBA, Salvador, Brazil), A.C.Camargo Cancer Center (São Paulo, Brazil) Hospital Dr. Rafael Angel Calderon Guardia (San José, Costa Rica) and Clínica Universitaria (Bogota, Colombia); and from CHU Rouen, Department of Molecular Genetics (Rouen, France).

Patients were informed about their inclusion into the registries and written informed consent was obtained from all participants during genetic counseling sessions.

### Nomenclature and classification of genetic variants

The recurrence of the identified variants was established by interrogating four databases (in their latest releases as of August 2024): the Leiden Open Variation Database (LOVD), the Universal Mutation Database (UMD), ClinVar and the Human Gene Mutation Database (HGMD). In addition, we also queried the local database of Rouen University Hospital in Rouen, France. Syntax of all variants was verified using Mutalyzer [38] using the current *PMS2* reference sequence (NM_000535.7). For PMS2 variant classification, the ClinGen InSiGHT Hereditary Colorectal Cancer/Polyposis Expert Panel Specifications to the ACMG/AMP Variant Interpretation Guidelines for PMS2 Version 1.0.0 were applied (https://cspec.genome.network/cspec/ui/svi/doc/GN139?version=1.0.0) with the exception of the evaluation of MMR assay results, which was performed according to the general ACMG/AMP sequence variant interpretation framework functional assay specifications [39].

### Cell lines

HeLa cells (RRID: CVCL_0030) were obtained from ATCC (ATCC CCL-2) and grown at 37° in DMEM medium with 10% FBS in a 5% CO_2_ atmosphere. HEK293T (RRID: CVCL_0063) cells were purchased at DSMZ German Collection of Microorganisms and Cell Culture (Braunschweig, Germany) and maintained in D-MEM containing 10% FCS and antibiotics/antimycotics. All cell lines were purchased less than one year before the initiation of the project and used in low passages. Mycoplasma tests were performed monthly to confirm their absence.

### Minigene constructs and minigene splicing assay

A cell-based RNA splicing assay using the two-exon pCAS2 minigene vector was used [40, 41]. Briefly, first the wild-type exons of interest (*PMS2* exons 8, 10, 12 and 14) and their flanking intronic sequences were amplified from the DNA of a control lymphoblastoid cell line and inserted into the intron of the pCAS2 minigene using *Bam*HI and *Mlu*I restriction sites. Since it was not possible to specifically amplify *PMS2* exon 12 due to the pseudogene *PMS2-CL*, the construct was corrected by site-directed mutagenesis to recapitulate the reference sequence of *PMS2* (Supplementary Table 1). The variants of interest were introduced by site-directed mutagenesis (Supplementary Table 1). Minigenes were transfected into HeLa cells using the FuGENE 6 Transfection Reagent (Roche Applied Science). Total RNA was extracted 24 h post-transfection using the NucleoSpin RNA Kit (Macherey-Nagel). The splicing patterns of the minigenes was analyzed by semi-quantitative fluorescent RT-PCR using 200 ng total RNA, specific minigene primers (Supplementary Table 1) and the OneStep RT-PCR kit (Qiagen), in 25 µl reactions. The number of PCR cycles selected for ensuring an amplification in the linear range was as follows: 30 cycles for minigenes carrying PMS2 exons 8, 10 or 12, and 34 cycles for those with exon 14. A fraction of the RT-PCR products was separated by electrophoresis on 2% agarose gels, gel-purified and then sequenced to determine the exact identity of each band. In addition, an aliquot from the remaining fraction was resolved by capillary electrophoresis on a 3500 Sequencer (Applied Biosystems). The resulting electropherograms were analysed using the GeneMapper v6.1 Software (Applied Biosystems) and peak areas were used to quantify the relative levels of each RT-PCR product.

### Analysis of patient’s RNA

Peripheral blood samples from three healthy donors and one patient heterozygous for *PMS2* c.1004A>G, all collected into PAXgene blood RNA tubes (Qiagen) were retrieved from the CHU Rouen Biobank [“Centre de Ressources Biologiques (CRB), CHU Rouen”], written consent having been obtained from all individuals. Total RNA was extracted from the four specimens by using the PAXgene Blood RNA kit. The obtained RNA preparations were used to assess possible variant-associated alterations in RNA splicing and allelic-specific expression (ASE). The splicing pattern of *PMS2* exon 10 in patient’s RNA was compared to that of control individuals by performing semi-quantitative fluorescent RT-PCR using 200 ng total RNA, primers in *PMS2* exons 6 and 11 (Supplementary Table S1), and the OneStep RT-PCR kit (Qiagen) in 25 µl reactions with 32 cycles of amplification. A fraction of the RT-PCR products was separated by electrophoresis on 2% agarose gels, gel-purified and then sequenced to determine the exact identity of each band. In addition, an aliquot of the remaining fraction was resolved by capillary electrophoresis on a 3500 Sequencer (Applied Biosystems) allowing to determine the relative amounts of the different RT-PCR products, as described above for the minigene splicing assay.

For allelic-specific expression (ASE), a SNaPshot quantitative primer extension assay was performed (ABI Prism SNaPshot) by using as template the patient’s RT-PCR products spanning PMS2 exons 6 to 11 described above. In parallel, a segment encompassing PMS2 exon 10 was amplified by PCR from the genomic DNA of the same patient by using the Multiplex PCR kit (Qiagen). 200 ng of genomic DNA, a forward primer in *PMS2* intron 9 (Supplementary Table 1) and a reverse primer in *PMS2* intron 10 in a final reaction volume of 25 µl and 30 cycles were used. Five µl of RT-PCR and PCR products were treated with one unit of Shrimp Alkaline Phosphatase (SAP, USB) and 8 units of Exonuclease I (Thermo Scientific) in SAP buffer in 10 µl final volume for 1 h at 37 °C. The reactions were terminated with 75 °C for 15 min. Two µl of purified RT-PCR and PCR products were subjected to primer extension reactions in a final volume of 10 μl using the SNaPshot Multiplex Kit (Applied Biosystems) and a reverse primer targeting the sequence immediately downstream PMS2 c.1004A>G (Supplementary Table S1). SNaPshot reactions comprised 25 cycles of primer extension (denaturation at 96 °C for 10 s, annealing at 50 °C for 5 s and elongation at 60 °C for 30 s). Next, the reactions were incubated with 1 unit of SAP at 37 °C for 1 h, and terminated at 75 °C for 15 min. Finally, the extension products were separated by capillary electrophoresis and analysed quantitatively by using a 3500 Genetic Analyzer (Applied Biosystems). SNaPshot signals from primer extensions on RT-PCR products (patient cDNA) were normalized to those obtained with PCR products (patient gDNA).

### Protein expression constructs

The pSG5 expression vector containing full-length human PMS2 cDNA was provided by Dr Bert Vogelstein (Johns Hopkins Oncology Center, Baltimore, MD, USA). The pcDNA3 expression vector containing the entire open reading frame of human MLH1 was a gift of Dr Hong Zhang (Huntsman Cancer Institute, University of Utah, Salt Lake City, UT, USA). Amino-acid positions in PMS2 refer to the 862 amino acid *PMS2* reference sequence (NP_000526.2).

Sequence variants of the PMS2 expression vector were generated using the Q5 site directed mutagenesis system (New England Biolabs, Ipswich, MA, U.S.A.) with the appropriate primers (Supplementary Table 2).

### PMS2 and MLH1 transient expression and protein extraction

MLH1- and PMS2-deficient HEK293T cells were transiently co-transfected with 2.5 µg of PMS2 and MLH1-vectors each and 20 mL of polyethyleneimine (1 mg/ml, “Max” linear, 40 kDa, Polysciences, Warrington, PA). Total and nuclear proteins were extracted as described previously [19].

### Protein steady-state expression analysis

Protein extracts were analyzed by SDS-PAGE and immunoblotting (using antibodies against PMS2 (anti-PMS2, E-19)), MLH1 (anti-MLH1, G168–728, BD Biosciences) and Actin (anti-beta-Actin, C2, from Santa Cruz Biotechnologies). Chemiluminescence signals (Immobilon, Millipore) were detected with an LAS-4000 mini camera (Fuji) and quantified using Multi Gauge v3.2.

### MMR activity

The MMR activity was scored in vitro as described [19, 26, 42]. Briefly, nuclear protein extract HEK293T cells (50 µg) was supplemented with 5 µg whole cell extract of HEK293T cells co-transfected with wild-type MLH1 and PMS2 vectors, or with wild-type MLH1 vector and the indicated PMS2 variant vectors. The extracts were incubated with 35 ng of DNA substrate containing a G-T mismatch and a 3’ single-strand nick at a distance of 83 bp in repair buffer for 15 min at 37 °C. Afterwards, the DNA substrate was purified and digested with EcoRV and AseI. The restriction fragments were separated in agarose gels and analyzed using GelDoc XR+ Imaging System and QuantityOne software (Bio-Rad). Repair efficiency (e) was calculated as: e = (intensity of bands of repaired substrate) / (intensity of all bands of substrate). Typical wild-type repair efficiencies ranged from 50-90%. The repair efficiency of PMS2 variants was analyzed in direct comparison to a wild-type protein that had been produced in parallel, and calculated as e(relative)=e(variant)/e(wild-type)*100. This assay has been extensively used in the functional analysis of 71 *MLH1* and *PMS2* variants [19, 20, 22, 24–26, 42–45], and its functional results have been shown to correspond to 31 available pathogenicity classifications (Supplementary Table 4).

### Bioinformatic analyses

#### Predictions of RNA splicing alterations

Predictions of variant’s direct impact on 3’ or 5’splice sites (ss) were obtained using Splice-Site-Finder Like (SSF-L) and MaxEntScan (MES), interrogated via the Alamut Batch v1.9 integrated software (Interactive Biosoftware, SOPHiA GENETICS, http://www.interactive-biosoftware.com) and via Splice AI using the Splice AI lookup web interface (https://spliceailookup.broadinstitute.org/), with a maximal distance of 500 nucleotides. Predictions of variants impact on Exonic Splicing Regulatory elements (ESR) were achieved by using QUEPASA (ΔtESRseq scores) and HEXplorer (ΔHZ_EI_ scores), both interrogated via Alamut Batch v1.9, as well as by using HAL (ΔΨ scores) via the corresponding online interface (http://splicing.cs.washington.edu/) as previously described [41, 46]. The Splicing Prediction Pipeline (SPiP) was also interrogated [47].

#### Conservation assessment

For conservation assessment of PMS2 amino acids, sequences for a comprehensive PMS2 alignment were retrieved using BLAST with the human PMS2 protein sequence (NP_000526.2) as query. The resulting hits were manually curated according to established procedures. Only one PMS2 sequence per organism was retained, and incomplete sequences were removed. MLH1 sequences accidentally included were identified by their highly conserved, C-terminal FERC sequence. By that procedure, 567 sequences from animals, 348 from fungi, 117 from embryophyta could be identified. An AlphaFold2 model of MLH1-PMS2 dimer was used for assessing the structural positions of the investigated residues [44, 48]. For assessing the conservation of a given residue in a specific sub-family of proteins, the corresponding position of the given residue in this sub-family was determined.

#### Protein prediction algorithms

MAPP prior probability values [49] for PMS2 variants were retrieved from http://hci-lovd.hci.utah.edu/home.php?select_db=PMS2_priors. AlphaMissense predictions [49] were retrieved using the Ensembl Variant Effect Predictor (VEP) site (https://www.ensembl.org/info/docs/tools/vep/index.html).

## Results

### Characterization of *PMS2* variants through clinical and population frequency analysis

We analyzed four *PMS2* missense variants identified in French and Latin American patients mapping to *PMS2* exons 8, 10, 12 and 14, respectively (Table 1). These *PMS2* variants have not yet been classified according to the MMR gene variant classification criteria [16, 23] and are currently listed as VUS in the Leiden Open Variation Database as well as in the ClinVar database, except for c.857A>G (p.(Asp286Gly)), p.1004A>G (p.(Asn335Ser)) and c.2395C>T (p.(Arg799Trp)) where conflicting classifications exist in ClinVar (VUS/Likely Benign/Benign) depending on the submitter, which likely is a consequence of different algorithms or lines of evidence applied by the submitters for evaluation of their individual contributions.

**Table 1:** *PMS2* variants analyzed in this study, gnomAD information and patient-related data.

PMS2 variant	Patient-related data								Further information (previous publications)	*ACMG classifi-cation based on clinical data*
Protein change/Nucleotide change/SNP identifier/gnomAD v4.1.0 Grpmax filtering allele frequency (corresponding ACMG criteria) and number of homozygotes	Identifier	Country	Gen-der	Carcinoma disease(s) (age at diagnosis in years)	Family data/criteria	MSI	IHC^3^	Further data ^1^		
p.Asp286Gly c.857A>G rs116788608 0.000124 (BS1) Homozygotes: 0	286-ARG-1	Argentina	m	CRC (40)	Bethesda		++00	MLPA: normal		*BP5*
	286-ARG-2	Argentina	f	Stomach (37); CRC (47)	Amsterdam I		///0			
	286-BRA-1	Brazil	m	Liposarcoma (38)	Not specific			*Additional VUS (126 genes tested):* WT1: c.1048 T > C; p.Cys350Arg		
	286-BRA-2=CM316	Brazil	f	Breast (39)	NCCN ^2^			BRCA1/2: no mutations. MLPA in BRCA1/2 normal		
	Li-14	France		CRC (42)		MSS	++++	One first degree relative with ovarian cancer (54 y); two distant relatives with CRC < 50 years.	See (Wang et al. 2020)	
	503989-623	France								
p.Asn335Ser c.1004A>G rs200513014 0.0002997 (BS1) Homozygotes: 0	335-URU-1	Uruguay	f	Colorectal adenoma (27); polyps in endometrium and ovary (22)	NCCN ^2^			MLH1, MSH2, MSH6, MUTYH, PMS1: no mutations		*PP4_moderate*
	335-BRA-1	Brazil	f	Ovarian germ cell (29); *CRC (36)*	*Bethesda*		//00	*Additional pathogenic variant:* MLH1 c.2004delA; p.Glu669Lysfs*114.		
Identified in 0.2% of index cases with HBOC^2^ syndrome in France	335-BRA-2=CM047	Brazil	f	Breast (42) Ovary (45)	NCCN ^2^			*Additional pathogenic variant:* BRCA1: c.5074+2 T > C. CHEK2: c.157 T > A. (VUS); MLPA in BRCA1/2 normal		
	335-ARG-1	Argentina	f	Ovary (39)	NCCN ^2^			MLPA: normal		
	Ro-23	France		Duodenal cancer (43)				First-degree relative with CRC (45)	See (Wang et al. 2020)	
	Ro-25	France		CRC (48)			+++/	First-degree relative with CRC (54)		
	Ro-30	France		CRC (61)		MSI-H	++00			
	Ro-93	France		CRC (42)			+++0			
	Ly-02	France		CRC (26)		MSI-H	+++0			
p.Ile679Thr c.2036T>C rs778251286 0.00000513 (PM2_supp) Homozygotes: 0	679-BRA-1	Brazil	f	CRC (50)	Bethesda	4	++++	No affected relatives		*Reach no criteria*
p.Arg799Trp c.2395C>T rs149202766 0.004805 (BA1) Homozygotes : 12	799-CRI-1	Costa Rica	f	Endometrium (31)	no cancer history in family		///0			*PP4_supp*
	799-COL-1	Colombia	f	Ovary (72)	NCCN (HBOC)^2^			*BRCA1/2, MLH1, MHS2, MSH6*: no pathogenic variants		
	799-COL-2	Colombia	f	Ovary (73)	NCCN (HBOC)^2^			*BRCA1/2, MLH1, MHS2, MSH6*: no pathogenic variants		
	799-COL-3	Colombia	f	Breast ductal (22)	NCCN (HBOC)^2^			*BRCA1/2, MLH1, MHS2, MSH6*: no pathogenic variants		
	799-COL-4	Colombia	f	Ovary (31)	NCCN (HBOC)^2^			*BRCA1/2, MLH1, MHS2, MSH6*: no pathogenic variants		
	799-COL-5	Colombia	f	Breast ductal infiltrating (35)	NCCN (HBOC)^2^			*BRCA1/2, MLH1, MHS2, MSH6*: no pathogenic variants		
	734388-623	France	f							

Variant descriptions confer to HGVS format and refer to the PMS2 reference cDNA (NM_000535.7) or protein (NP_000526.2) sequences and have been checked using Mutalyzer Name Checker. ^1^ Except otherwise stated, no other variants have been identified in MMR genes in these patients by sequencing. ^2^ NCCN: National Comprehensive Cancer Network Guidelines; HBOC: Hereditary breast and ovary cancer. ^3^ Immunohistochemical protein expression for MSH2-MSH6-MLH1-PMS2; +: normal expression; 0: no expression; /: not tested or not conclusive. n.a. not applicable.

The first variant, c.857A>G (p.(Asp286Gly)), has been identified in six unrelated carriers (four from Latin America and two from France), three of these with colorectal cancer diagnosed before the age of 50 years, including one case with a tumor showing loss of PMS2 as determined by IHC, and one with loss of MLH1-PMS2. These data could reach a PP4_moderate criterion according to ClinGen InSiGHT Specifications to the ACMG/AMP Variant Interpretation Guidelines Version 1 (https://cspec.genome.network/cspec/ui/svi/doc/GN139?version=1.0.0.), but the identification of this variant in 2 patients with CRC showing MSS and/or normal IHC could also reach a BP5 criterion. This led us to consider no clinical criteria for the classification of this variant.

The second variant, *PMS2* c.1004A>G (p.(Asn335Ser)), was found in four unrelated carriers from Latin America, of which one has Lynch syndrome due to a pathogenic *MLH1* variant and one is positive for hereditary breast cancer (*BRCA1* pathogenic variant). It was also detected in five unrelated patients with gastrointestinal tumors in France, three of which with a CRC tumor showing MSI and/or loss of PMS2, allowing to quote a PP4_strong criterion. In addition, c.1004 A > G (p.(Asn335Ser)) was identified in ~0.2% HBOC syndrome-suspected probands from France. The third variant, *PMS2* c.2036T>C (p.(Ile679Thr)), was identified in a single CRC patient from Brazil with age at diagnosis of 50 years. The fourth variant, *PMS2* c.2395C>T (p.(Arg799Trp)), was found in seven cancer patients (from Latin America and France), either with endometrial, breast or ovarian tumors. The loss of PMS2 expression on the endometrium tumor quotes a PP4_supporting criterion.

Three variants (p.(Asp286Gly), p.(Asn335Ser) and p.(Arg799Trp)) feature population frequencies suggestive of benignity (BS1), with p.(Arg799Trp) additionally having a large number of reported homozygotic carriers who are not affected of CMMRD, which would be the case if this variant were pathogenic. In contrast, p.(Ile679Thr) is a rather rare variant, compatible with pathogenicity (PM2_supp) (Table 1).

### Effect of variants on RNA splicing

The potential impact on RNA splicing was evaluated using several in silico prediction tools. Two *PMS2* variants (c.857A>G and c.1004A>G) were predicted to negatively affect splicing regulation by three and one algorithm, respectively (Table 2), while the other two (c.2036T>C, c.2395C>T) were predicted not affect RNA splicing signals.

**Table 2:** In silico predictions of potential variant-induced RNA splicing alterations.

*PMS2* variant	Exon	SSF-L (∆%)	MES (∆%)	QUEPASA (∆ESRseq)	HEXplorer (∆HZEI)	HAL (∆Ψ)	SPiP	Splice AI (threshold=0.2) Score (and distance relative to variant position)				Interpretation
		*Threshold: -5%*	*Threshold: -15%*	*Threshold: -0.5*	*Threshold: -15*	*Threshold: -15*		3’ss Loss	5’ss Loss	3’ss Gain	5’ss Gain	Predicted effects on RNA splicing signals
c.857A>G p. (Asp286Gly)	8	0	0	**-2.24**	**-41.28**	-3.4	**0.45**	0.06 (53)	0.02 (-46)	0	0	Negative effect on splicing regulation
c.1004A>G p. (Asn335Ser)	10	0	0	**-1.69**	-10.9	0	0.122	0.01 (-12)	0	0	0	Negative effect on splicing regulation
c.2036T>C p. (Ile679Thr)	12	0	0	0	18.17	0.7	0.038	0.01 (-57)	0	0	0	None
c.2395C>T p. (Arg799Trp)	14	0	0	-0.39	-2.74	-2.2	0.02	0.01 (76)	0	0	0	None

Values shown in bold are indicative of potential splicing defects.

**Fig. 1:**
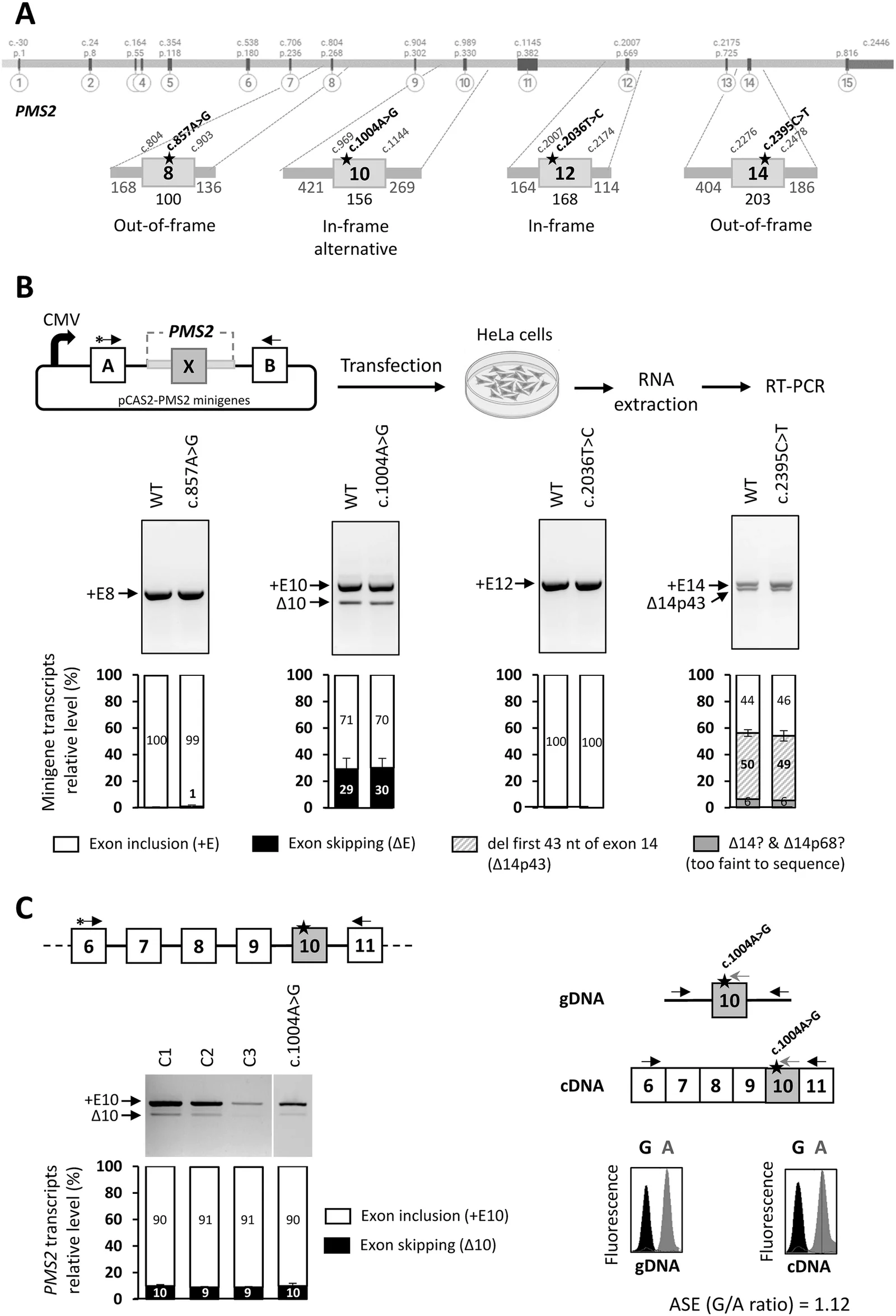
RNA splicing analyses. **A** Structure of the *PMS2* gene and relative position of the four variants analyzed in this study. **B** Minigene splicing assays. RT-PCR primers are represented by black arrows (5’FAM modification indicated by *). The graphs show the average of 3 independent experiments. **C** Analysis of RNA extracted from the peripheral blood of a patient heterozygous for *PMS2* c.1004A>G. Left panel, RT-PCR results obtained with RNA from the patient analyzed in parallel of equivalent samples from three control individuals (C1, C2 and C3). The graph shows the average of 2 independent experiments. Right panel, results from allele-specific expression (ASE) analysis using the SNaPshot primer extension method. The position of the primer used in the extension reaction is shown by the grey arrow. The fluorochrome electropherogram images show the outcome of 1 out of 2 experiments with similar results. The identity of the peaks (G and A) was converted to reflect the sequence in the sense strand. The ASE value indicates the level of expression of the variant allele (G) relative to the WT allele (A) calculated by normalizing the fluorescence obtained with the cDNA template with that obtained with gDNA. WT, wild-type.

For experimental verification, we performed cell-based minigene splicing assays in which we compared the splicing pattern of wild-type and mutant PMS2 minigenes transiently expressed in human cells. RT-PCR analysis of the minigene-transcripts revealed that none of the variants had a major impact on splicing in these conditions suggesting that they are all authentic missense alterations (Fig. 1A, B).

For further verification, we directly analyzed RNA from one heterozygous carrier of *PMS2* c.1004A>G. RT-PCR analysis of this sample, in parallel to equivalent samples from three control individuals, revealed a normal *PMS2* splicing pattern, and allele-specific expression analysis showed a balanced *PMS2* expression in this carrier (Fig. 1C).

### Protein stability and MMR activity of the variants

**Fig. 2:**
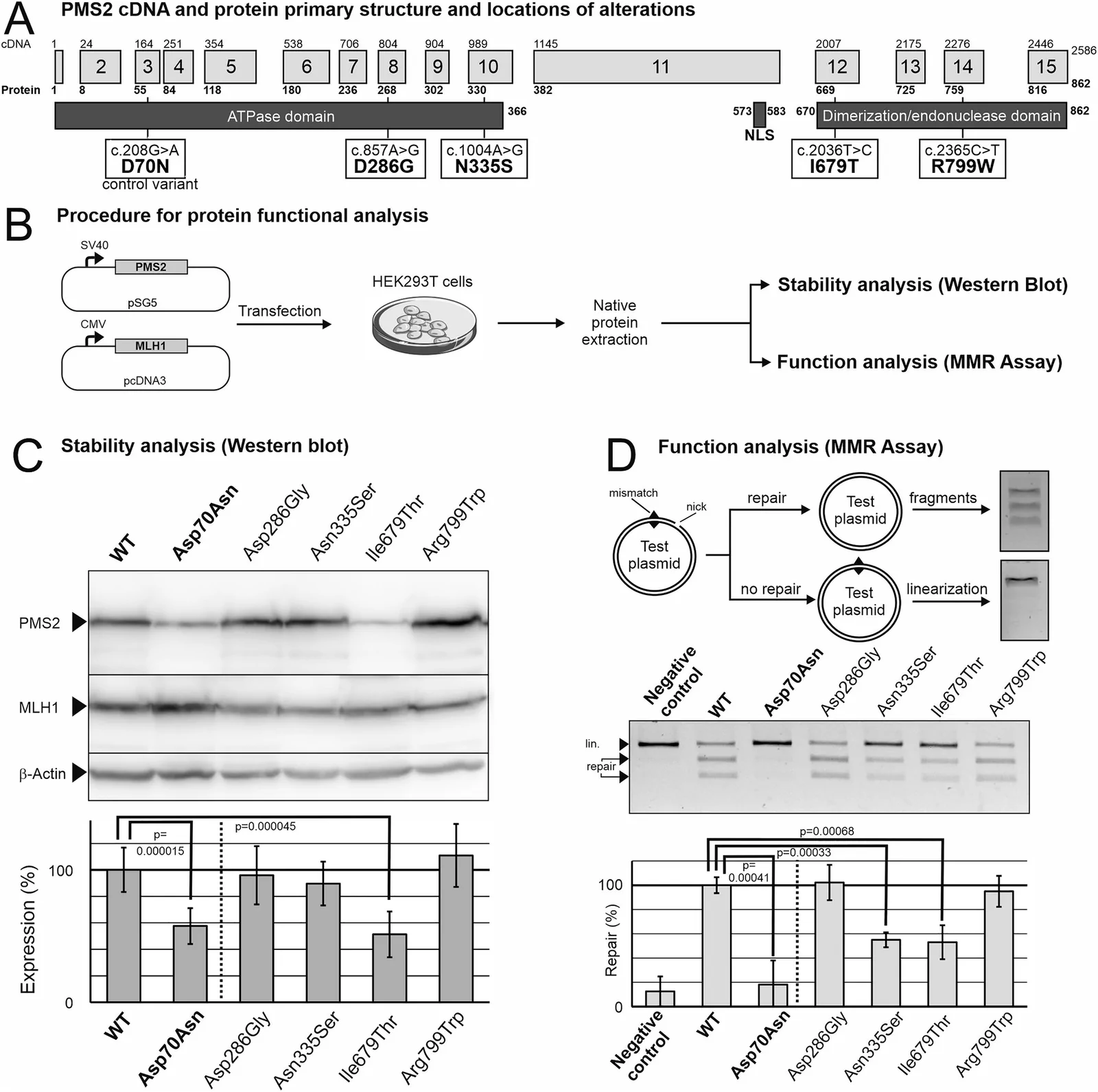
Protein functional analysis. **A** Schematic representation of the primary structure of PMS2 on the cDNA- and protein-level including the locations of the different missense variants investigated in this study. We tested one pathogenic control variant from the ATPase domain (p.Asp70Asn) and the four VUS of interest. intron-exon structure and annotations of functional motifs in the protein are given. **B** Schematic representation of the procedure for protein functional investigation. **C** Stability analysis by expression and western blotting. Expression plasmids for MLH1 and PMS2 (and its variants) were co-transfected in HEK293T cells. After 48 h, whole cell extracts were prepared and analyzed using SDS-PAGE and immunoblotting. β-Actin detection served as loading control. Expression levels of several experiments (3-8) in comparison to wildtype (%) were determined. Bars correspond to standard deviations. Controls and test variants are separated by a dashed line in the bar diagram. **D** Functional analysis by MMR (mismatch repair) assay. A test substrate with a G-T mismatch (indicated by triangles) within an *Eco*RV restriction site and a single-strand break (“nick”) directing repair to the open strand was incubated as detailed in Materials and Methods with nuclear extract of MLH1-PMS2-deficient HEK293T cells (50 µg) and complemented with extract containing the MLH1-PMS2 wildtype or variant as indicated (5 µg). After 15 min at 37 °C, the plasmid was isolated and analyzed by restriction digestion and agarose gel electrophoresis to test the functionality of the *Eco*RV restriction site. The appearance of two lower-weight fragments indicates repaired plasmid, while the upper band stems from unrepaired plasmid. Several independent assays were performed, and average repair values and standard deviations are shown in the bar diagram. Controls and test variants are separated by a dashed line in the bar diagram.

Two variants (p.Asp286Gly and p.Asn335Ser) map to the ATPase domain of PMS2, the other two (p.Ile679Thr and p.Arg799Trp) are located within PMS2 C-terminal domain which is responsible for dimerization of PMS2 with MLH1 and contains the endonucleolytic activity (Fig. 2A). Wild-type MLH1-PMS2 and variant PMS2 vectors were expressed in HEK293T cells [51], followed by analysis of MLH1/PMS2 protein stability and function (Fig. 2B). As negative control for function, we used samples without MLH1-PMS2 and additionally PMS2 p.Asp70Asn, a variant of a highly conserved residue that has been shown before to be non-functional because it inactivates binding of the nucleotide in the ATPase pocket of PMS2 [52], since it has been used before as a known MMR-defective control variant [22].

All variants produced PMS2 protein to levels equivalent to WT except for p.Ile679Thr which resulted in significantly reduced PMS2 expression (*p* < 0.001) to a level similar to the that of the non-functional control variant p.Asp70Asn (Fig. 2C). In contrast, MLH1 expression levels were on average not affected by the alterations in PMS2 (Fig. 2C and Supplementary Fig. 1).

Subsequently, all variants were tested in an in vitro DNA-MMR-assay that has been used extensively before by us and others to characterize variants in MLH1 and PMS2. Since MLH1 and PMS2 require to dimerize to form a functional MMR complex, it is always the activity of this complete functional complex that is determined, making it possible to analyze the functional impact of both MLH1 and PMS2 variants. This assay is currently not listed in the ClinGen InSiGHT Hereditary Colorectal Cancer/Polyposis Expert Panel Specifications to the ACMG/AMP Variant Interpretation Guidelines for PMS2 Version 1.0.0, (https://cspec.genome.network/cspec/ui/svi/doc/GN139?version=1.0.0). However, the assay has been used to functionally characterize 71 human variants (69 MLH1 and 4 PMS2) [19, 20, 22, 24–26, 42–45]. Of these, 31 variants (28 MLH1 and 3 PMS2) were in the meantime classified as either (likely) pathogenic [21] or (likely) non-pathogenic [10]. All 31 variants were correctly interpreted using this assay (Supplementary Table 3A). According to the ACMG/AMP sequence variant interpretation framework, the assay therefore has functional odds of pathogenicity of 10,0 and 0.048 (Supplementary Table 3B), corresponding to the evidence strength classifications PS3_moderate and BS3, respectively [39].

The MMR activity of the pathogenic variant control p.Asp70Asn was abolished, while both the first and the fourth variants, p.Asp286Gly and p.Arg799Trp, were indistinguishable from wild-type, which validates a BS3_moderate criterion and should allow us to consider these variants as likely benign variant (class 2). In contrast, both the second and the third variant, p.Asn335Ser and p.Ile679Thr, showed a significant, strong defect in MMR activity (*p* < 0.001), although less severe than that of p.Asp70Asn (Fig. 2D).

### Structured analysis of residue conservation and possible structural consequences

**Fig. 3:**
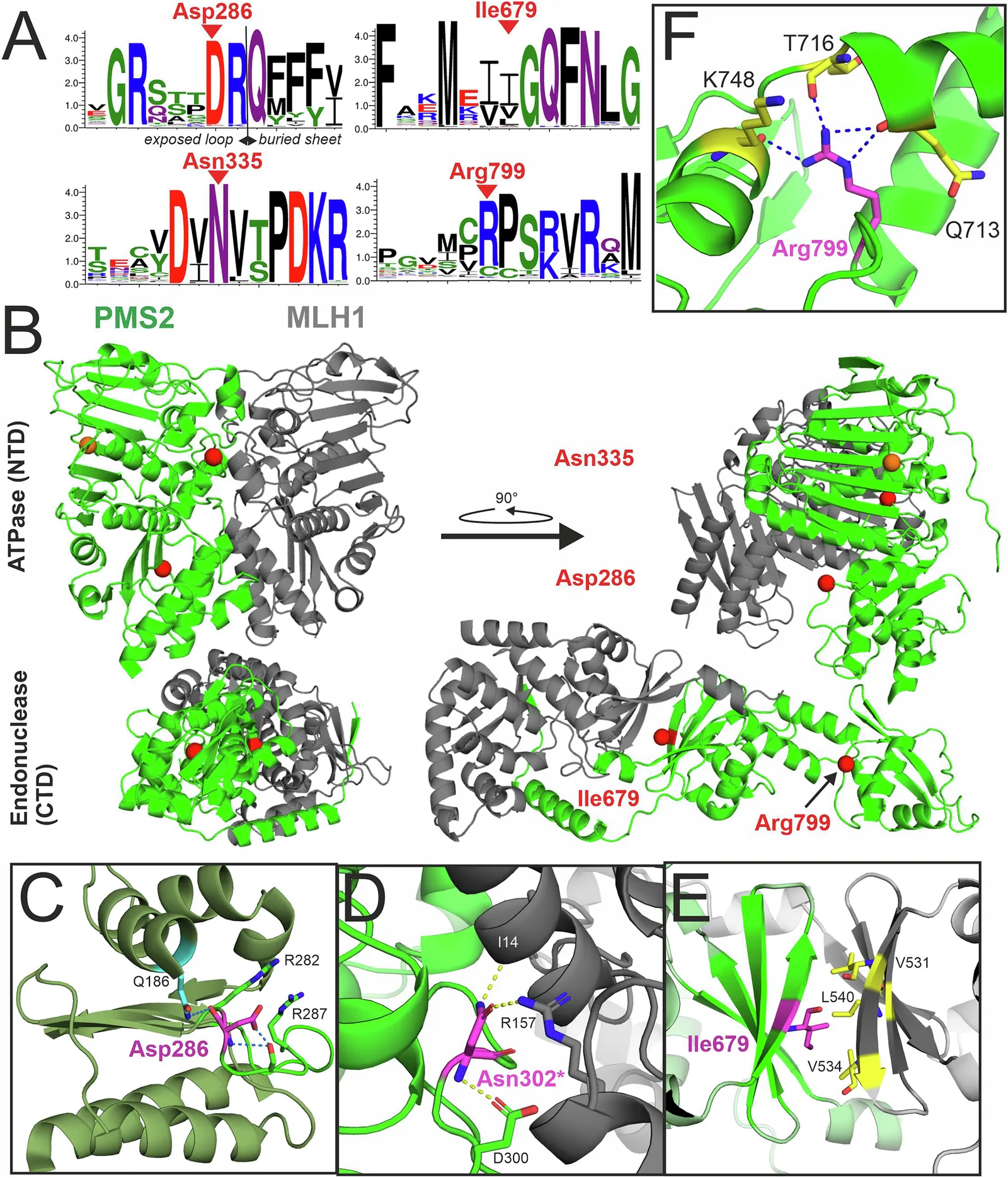
Conservation and structural role of the variant PMS2 residues. **A** Conservation of the residues affected by alterations in WebLogo representation. The affected residue is shown by a rot dot underneath the diagram. **B** AlphaFold2 model of a complete MLH1-PMS2 heterodimer showing the ATPase in the N-terminal domain (NTD) and the endonuclease located in the C-terminal domain (CTD). Both structured domains are connected by an unstructured linker region (not shown). For marking the positions of the affected residues, their respective Cα atoms are represented as red balls. **C** Detail of the unstructured surface loop in which Asp286 is located is colored in brighter green. Together with the conserved neighboring residues R282 and R287, Asp286 forms a hydrophilic loop surface. By its main chain carbonyl function, it forms a contact to Q186 which contributes to arresting the loop in its conformation. **D** N302 is the residue that corresponds to human PMS2 Asn335. The image shows its role in formation of the N-terminal dimer using the bacterial MutL structure (1b63). The subunit corresponding to PMS2 is shown in green, the other subunit corresponding to MLH1 is shown in grey. N302 forms a side-chain interaction to the side-chain of R157 of the opposing subunit. It also contacts the main chain of I14 of the opposing subunit. Within its own subunit, the conformation of the loop is supported by an interaction of D300 with N302, explaining the high conservation of this neighboring aspartate. **E** Structure detail of Ile679 whose side chain contributes to hydrophobic interactions establishing (with hydrophobic MLH1 residues V531, V534 and L540, shown in yellow) the core of the C-terminal MLH1-PMS2 dimerization interface. **F** Structure detail of Arg799 (purple) located within the interieur of the PMS2-CTD and engaging in polar contacts with main chain atoms of three distant residues (yellow). *E.coli N302 corresponds to human Asn335.

Assessment of the biochemical role of an amino acid within the protein structure can explain, on a molecular level, functional defects of a missense variant [43–45]. Vice versa, it is desirable to support the analysis of a substitution by bioinformatics analyses and structural evaluation. MAPP predictions are being used as prior probability values for pathogenicity [16, 53], and the more recent AlphaMissense algorithm also provides pathogenicity estimates [50] (Table 3). However, while the prediction of protein folding has recently progressed markedly with the introduction of AlphaFold2 [48], prediction of the consequences of individual substitutions still represents a challenge [54, 55]. Therefore, manual individual inspection of a substitution may provide additional evidential value. In order to place this individual inspection on a more reproducible, defined basis, we devised a scheme of questions (“ConStruct Assessment Form”, Supplementary Table 4) tailor-made for the analysis of MutL proteins, which simultaneously provides individual analytical steps and allows to document their results and thereby provides a trackable procedure for this assessment. The ConStruct procedure is explained in more detail in the appendix of the Supplementary Data. Using the procedure, we could confirm that, Ile679 and Asn335, although less or similarly conserved like Asp286 and Arg799 (Fig. 3A), are highly relevant for N-terminal and C-terminal dimerization (Fig. 3D, E and Supplementary Table 4). In contrast, an effect on structure or function remains possible, but seems less likely for Asp286 and Arg799 (Figs. 3C, F and Supplementary Table 4), consistent with the biochemical results.

**Table 3:** Summary evaluation of conservation, structure and experimental results and application of classification criteria.

PMS2 protein variant (NP_000526.2)	MAPP/PP2 Prior probability of pathogenicity AlphaMissense prediction	ACMG classification of MAPP/PP2 prior	DeMaSk score	Conservation score^1^				Evaluation of conservation and structure	Criteria applied based on experimental Results on RNA and protein level^2^	Interpretation on functional level and classification^2^	Variant classification^2^
				PMS2	MLH1	MutL	Average				
p.Asp70Asn	0.7383	PP3	-0,31	**9**	9	9	**9**	Absolutely conserved in MutL, known role in function (ATP binding)	*n.a*.	Dysfunctional control variant (22;51)	n.a. (control variant)
p.Asp286Gly	0.7635 0.6286	PP3	-0,25	**9**	6	7	**7**	Only conserved in PMS family proteins, role in formation of hydrophilic loop surface. Although non-conservative exchange, hydrophilic surface of loop is maintained by charged neighbor residues.	*BS3*	Functional	BS1, BS3, BP5, PP3: **Benign**
p.Asn335Ser	0.6062 0.585	Reaching no criteria	-0,30	**9**	9	9	**9**	Absolutely conserved in MutL, known role in function (N-terminal dimerization)	*PS3_moderate*	Function compromised	BS1, PP4_moderate, PS3_moderate: **VUS**
p.Ile679Thr	0.5105 0.182	Reaching no criteria	-0,24	**8**	9	7	**8**	Hydrophobic residue absolutely conserved in MLH1 and PMS family. Contributes to hydrophobic core of dimerization interface.	*PS3_moderate*	Function compromised	PM2_supp, PS3_moderate: **VUS**
p.Arg799Trp	0.8917 0.7692	PP3 _moderate	-0,34	**9**	7	5	**7**	Only conserved in PMS family proteins. Surface residue.	*BS3*	Functional	BA1, BS3, PP4_supp, PP3_moderate: **Benign**

^1^ Conservation assessed with the alignments of the respective protein families by ConSeq (see Materials and Methods).

^2^ Using ClinGen InSiGHT Hereditary Colorectal Cancer/Polyposis Expert Panel Specifications to the ACMG/AMP Variant Interpretation Guidelines for PMS2 Version 1.0.0 (https://cspec.genome.network/cspec/ui/svi/doc/GN139?version=1.0.0)

## Discussion

The available clinical, family history and molecular data were insufficient to clinically classify the four *PMS2* variants. We therefore performed functional investigations and in silico analyses. The *in cellulo* experiments revealed that, in contrast to certain bioinformatics predictions, none of the variants had a negative effect on RNA splicing. Protein functional assays identified reduced MMR activity for p.Asn335Ser, and decreased protein stability and MMR activity for p.Ile679Thr. Application of the ConStruct scheme in search for molecular confirmation of these findings suggested a deleterious effect of p.Asn335Ser because of its intimate involvement in transient, N-terminal dimerization, and for p.Ile679Thr since it introduces a hydrophilic, bulky residue in the hydrophobic dimerization interface. For the other two affected residues, there was no strong evidence in conservation or structure that suggested that the alterations confer a loss of protein function. These findings support the utility of ConStruct as a complementary tool for variant interpretation.

Notably, both variants had incomplete defects in MMR, for which the following explanations can be found: Asn335 belongs to a framework of residues performing ATP-dependent N-terminal dimerization which are absolutely conserved in MutL proteins (Table 3, Supplementary Fig. 2) [56]. Since ATP binding causes N-terminal dimerization and this enables hydrolysis, a defect in the ATPase cycle can be expected. However, the alteration affects only one of four interactions relevant during N-terminal dimerization (Supplementary Fig. 2), therefore a residual activity is likely, possibly in the unaffected MLH1 subunit, which displays a greater ATPase activity than the PMS2 subunit [52]. A recent study of p.Asn335Ser concluded that this variant confers no defect in MMR [57]. This discrepancy may arise from methodological differences, specifically two key aspects: (i) the ATPase measurements of D’Arcy et al. were performed with the isolated NTD of PMS2 and therefore could not reflect the proper MLH1-PMS2 ATPase cycle that may include an N-terminal dimerization of MLH1 and PMS2. (ii) The sensitivity (relative signal intensity, dynamic range) of the MMR assay performed by D’Arcy et al. is much lower than the MMR measurements performed in this study, which likely rendered the partial MMR defect of p.Asn335Ser undetectable in the work of D’Arcy et al. Additionally, our finding fits well with the observation that PMS2 hydrolysis mutants also display MMR activities reduced by 50% [52]. Taken together, we assume that p.Asn335Ser confers a defect in the ATPase activity which results in a 50% decline in overall repair activity.

The intermediate effect on expression and activity of variant p.Ile679Thr is also likely caused by an incomplete effect on dimerization, since the substitution to the hydrophilic threonine will likely disturb dimerization resulting in destabilization of PMS2 but not fully abolish dimerization, since this is cooperatively established by a rather large protein surface (Fig. 3E).

The incomplete defect of both variants raises the question if the intermediate degree of dysfunction will cause a clinically relevant cancer predisposition phenotype. We and others have before described that incomplete defects in *MLH1* missense variants correlate with less severe clinical phenotypes [19, 29]. The phenotype of carriers of *path_PMS2* variants is already less pronounced than that of *path_MLH1* carriers, therefore it is unclear how strong the tumor predisposition of incomplete PMS2 defects will be, since the correlation of PMS2 variant activities with clinical phenotypes has not yet been established. The observed functional defects of roughly 50% loss of activity can be expected to translate into a weaker clinical phenotype in p.Asn335Ser and p.Ile679Thr carriers than in *path_PMS2* carriers. Therefore, in order to make meaningful conclusions, further investigations are required to generally establish the clinical relevance of path_PMS2 alterations.

We have also investigated two further variants of conserved residues for which we did not identify functional defects (p.Asp286Gly and p.Arg799Trp). Interestingly, both feature higher probabilities of pathogenicity than the defective variants both in MAPP and in AlphaMissense predictions (Table 3) [50]. In contrast, both deficient variants (p.Asn335Ser and p.Ile679Thr) were predicted to have less severe consequences in silico (Table 3). A possible explanation is that in silico predictions underestimate substitution effects when they are located in protein interaction sites that are not occupied when only structural data of the solitary protein are analyzed. Anyway, discrepancies in genotype-phenotype predictions for AlphaMissense have been observed before, and accurate predictions remain a challenge [54]. These observations suggest that the manual investigation as performed using the ConStruct assessment may in certain cases better reflect functional consequences of substitutions (Supplementary Table 4). However, while ConStruct therefore may provide additional evidential value for pathogenicity assessment, a comprehensive validation potentially enabling its implementation in an evaluation framework still needs to be performed.

In conclusion, we have provided comprehensive clinical and functional data for four unclassified *PMS2* genetic variants. The structured analysis of conservation and structure (ConStruct) supported that two variants retained normal function, while two had a significant, but incomplete loss of function, which could be explained on a molecular level and probably translates in a cancer predisposition less severe than observed in *path_PMS2* carriers. We suggest considering p.Asp286Gly and p.Arg799Trp as benign variants. While both p.Asn335ser and p.Ile679Thr had compromised biochemical function, the evidence in summary did not suffice to make a conclusion (Table 3).

## Supplementary information

SUPPLEMENTARY MATERIAL

## Data Availability

All data are included in this article or its supplementary information.
