# Supplementary material for: Analysis of structure and conservation for supporting functional evaluation of PMS2 missense variants
Source: Eur J Hum Genet. 2026 Mar 18;34(9):1342–54. doi: 10.1038/s41431-026-02072-3 (PMC13550630; doi:10.1038/s41431-026-02072-3)
Supplement: Supplementary file 1 [file 41431_2026_2072_MOESM1_ESM.pdf]

SUPPLEMENTARY MATERIAL

Nicholas Zeuzem[1], Manon Quilan[2], Mev Dominguez-Valentin[3], Stéphane Baert-Desurmont[4], Madleen Horlacher[1], Adriana Della Valle[8], Patricia Esperon[8,9], Florencia Neffa[8], Taisa Manuela Bonfim Machado-Lopes[10], Ivana Lucia de Oliveira Nascimento[10,11], Maria Betânia Pereira Toralles[10], Thaís Ferreira Bomfim-Palma[10], Walter Hernan Pavicic[12], Carlos A. Vaccaro[12], Florencia Spirandelli[13], Carlos Santamaria-Quesada[14], Geiner Jimenez[15], Felipe Vaca-Paniagua[16,17,18], Sandra Perdomo[19], Juan Javier López Rivera[20], Giovana Tardin Torrezan[21], Dirce Maria Carraro[21], Angela Brieger[1], Hubert Serve[5,6,7], Alexandra Martins[2], and Guido Plotz[1]

Analysis of structure and conservation for supporting functional evaluation of *PMS2* missense variants

Supplementary Figure 1

MLH1 expression in combination with PMS2 variants.

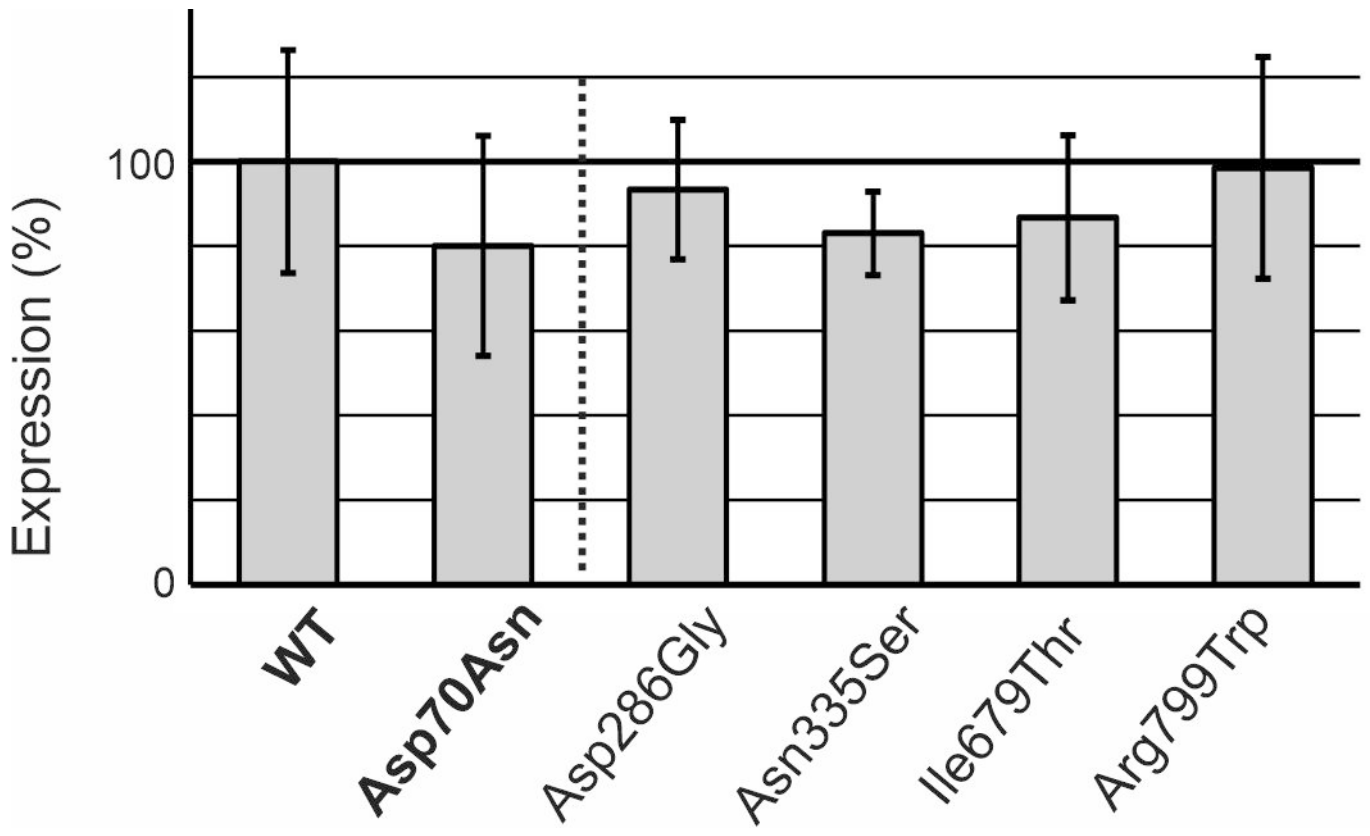

In parallel to PMS2 protein expression, MLH1 expression levels (see Figure 2C) of several experiments (3-8) were quantified in comparison to wildtype (%). Bars correspond to standard deviations. Controls and test variants are separated by a dashed line in the bar diagram.

Supplementary Figure 2

A conserved polar side-chain interaction network for N-terminal dimerization.

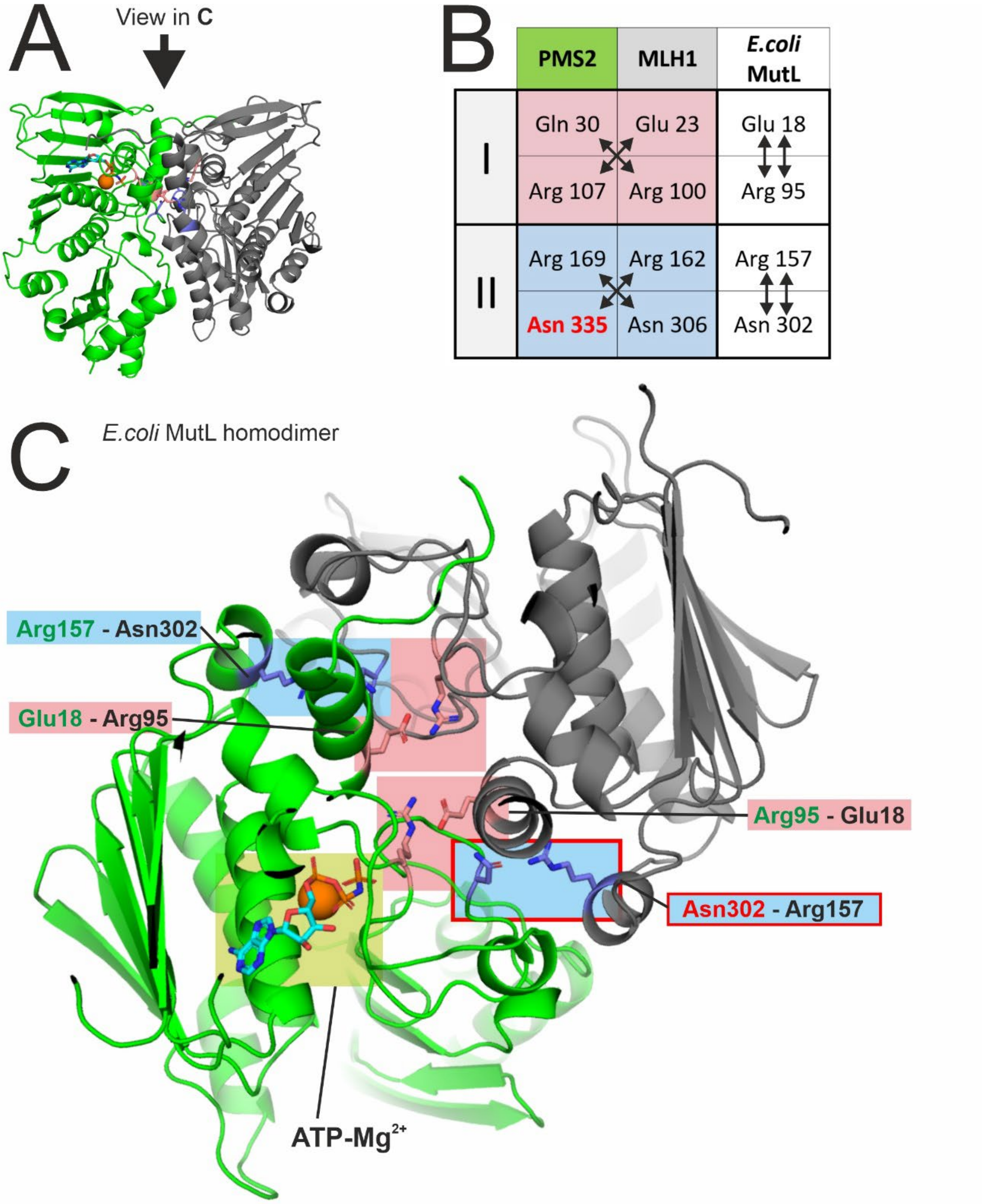

The figure shows the N-terminal biological dimer of bacterial MutL (*E.coli*; pdb1b63). N-terminal dimerization relies on four polar side chain interactions of highly conserved residues, one pair being formed with Glu18-Arg95 and the other with Arg157-Asn302. **A**. Side view of the MutL NTD with the bound non-hydrolyzable ATP analogue AMP-PNP in ball-and-stick presentation with an Mg^{2+} ion and the residues performing the inter-dimer polar contacts in ball and stick representation. When seen from the top (see arrow), the view is as shown in C. **B**. Overview of the side-chain interaction network in human MLH1-PMS1 and *E.coli* MutL. **C**. The four side-chain-interactions of the two residue pairs that bridge the two NTDs are boxed with blue and red shading. The residue numbering in the corresponding human MLH1-PMS2 protein is given in the boxes. Alteration of PMS2 Asn335 (red) will result in loss of one of these four interactions.

Supplementary Figure 3

Sequence conservation projected on PMS2 protein surface

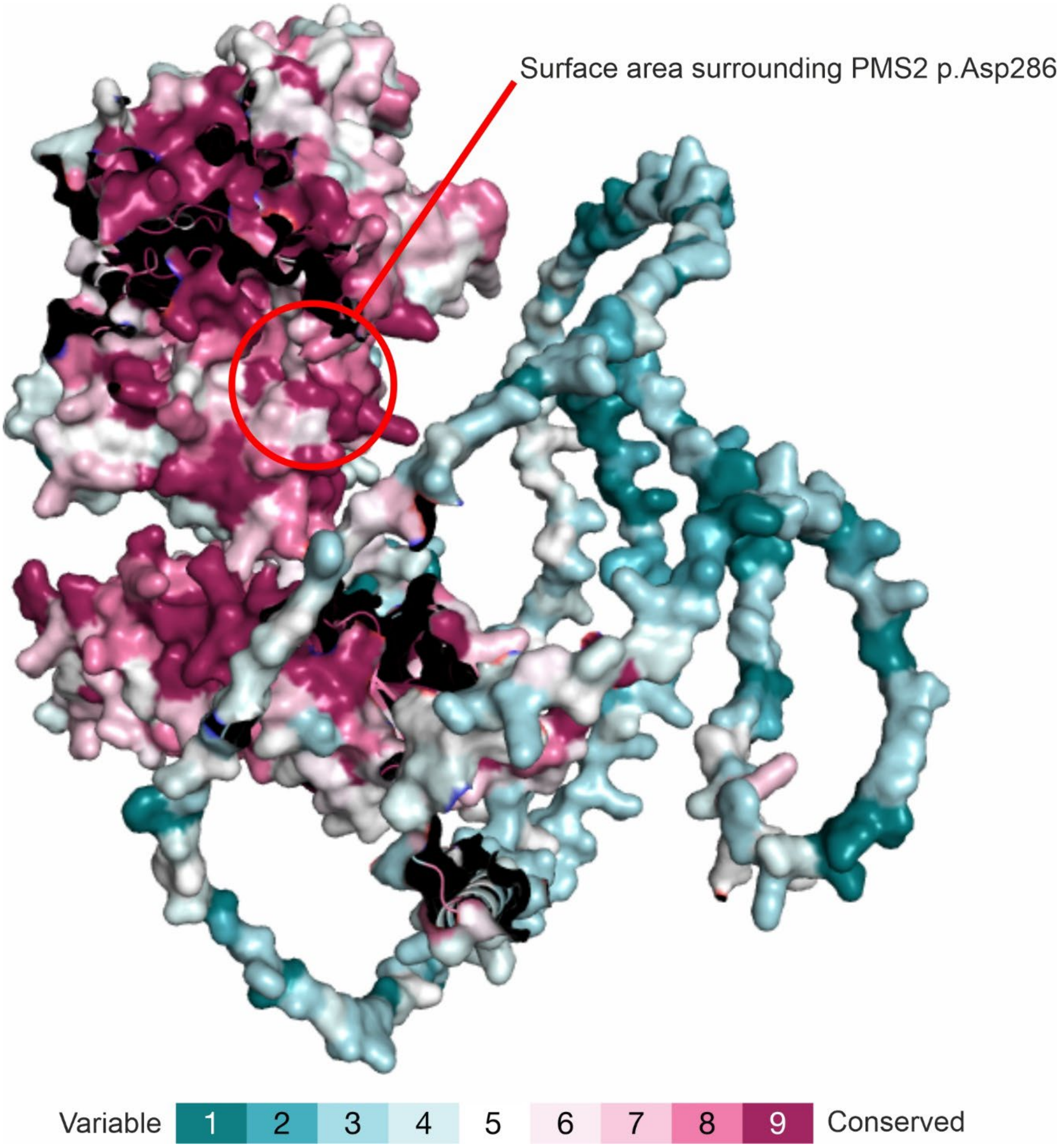

Sequence conservation of PMS2 proteins was scored and projected on the surface of the PMS2 protein model generated by AlphaFold2. The red circle indicates the surface area surrounding the p.Asp286 residue in the NTD of PMS2.

APPENDIX

Explanations on the ConStruct assessment (Supplementary Table 2)

The analysis begins with conservation scoring, first giving the number of sequences used for the alignment that underlies this analysis, and the residues corresponding to the analyzed residue in other MutL family branches (A1). Since MLH1-PMS2 represents a heterodimer that has evolved from a homodimeric ancestor (bacterial MutL), it is of interest to test if a given residue is conserved in the whole MutL family or only in specific sub-families (A2). A conservation in the whole MutL family indicates that the residue is part of a key structure vital for all MutL family members and therefore fundamental for MutL function (ubiquitously conserved):

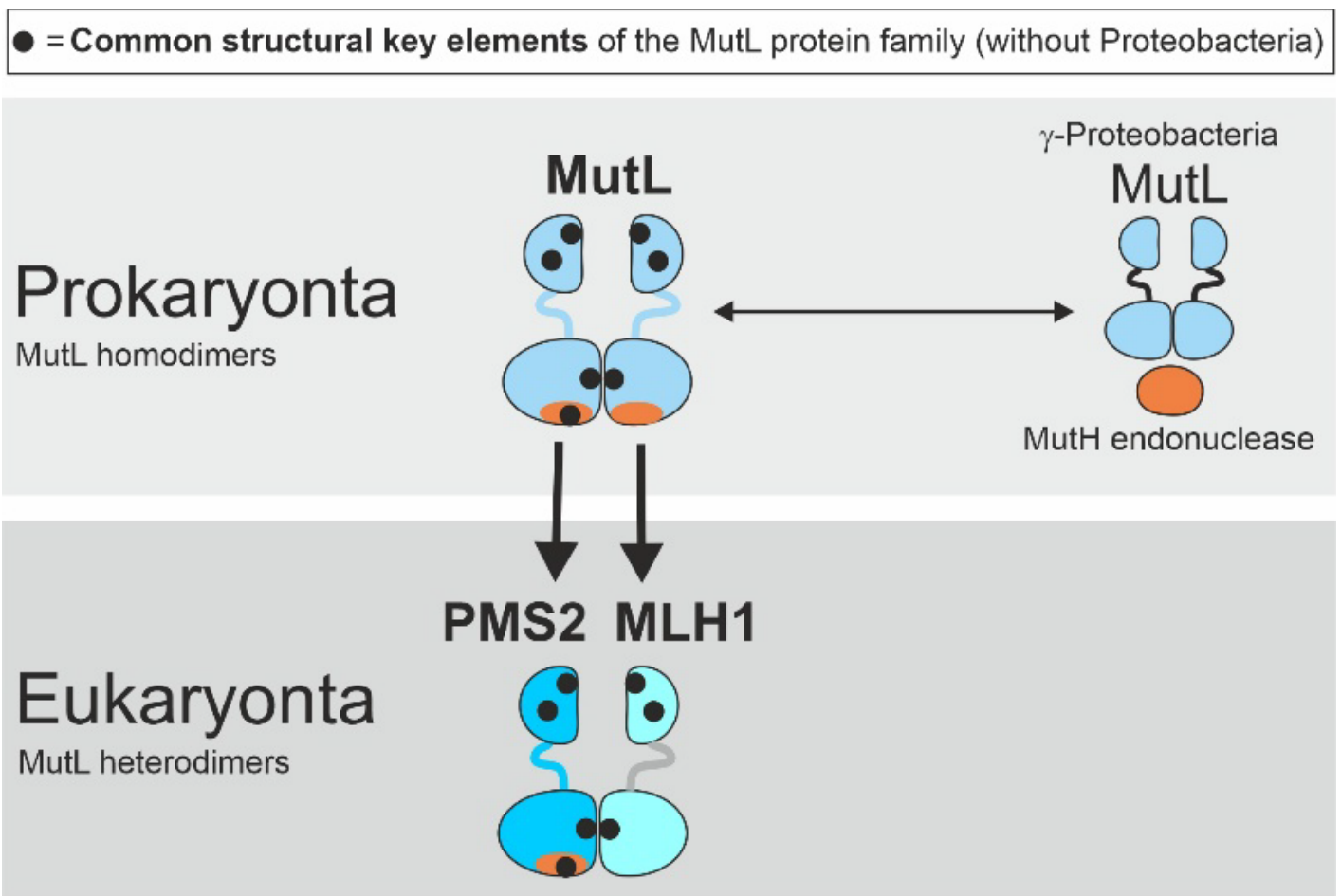

This analysis therefore provides an additional layer of information exceeding summary overall conservation scores. Within the bacterial MutL family, we excluded MutL proteins from γ-Proteobacteria since these do not encode a MutL endonuclease (Putnam 2016).

Conservation analysis also incorporates an evaluation if a hydrophobic residue is conserved in the position, which may falsely reduce the conservation scoring since these are potentially interchangeable in a hydrophobic surrounding (A3).

A large number of structures is available for MMR proteins, as well as a range of AlphaFold2 predictions from diverse organisms. The evaluation of the structural role therefore begins with a decision for structures to be used for the analysis. For that, it is of interest to verify if the roles and positions of the residue in question are comparable in the available structures (B1). If this is the case, this reinforces reliability of the structures and the ensuing analysis. In contrast, differences in structures may indicate a poor reliability in the investigated region and prompt the question which structure best represents the biological situation.

Subsequently, it is evaluated if the residue is located within a known functional region of the protein, which elevates the potential to interfere with function (B2).

Since protein regions showing secondary structure are more sensitive to alterations, this is interrogated in section B3.

A major part of the stability of a protein derives from the formation of a hydrophobic cores and side chain polar interactions in the interior of the protein (Dill 1990; Nick Pace et al. 2014). Hence, it the next two sections investigate the potential role of such interactions (B4 and B5).

Finally, in case of residues located on the protein surface, these may either also contribute to formation of a hydrophilic protein surface, to surface stability by creation of salt bridges or may even contribute to formation of an interaction area. This is investigated in B6.

Application of the ConStruct assessment on four variants (Supplementary Table 2)

We first scored conservation in a comprehensive alignment of PMS2 orthologs (**Table 3, Figure 3A**). All investigated residues were highly conserved in the PMS family, three achieving the highest scores (9), while p.(Ile679Thr) achieved a slightly lower score of 8 (**Supplementary Table 4** *A2*). This variant also features the lowest MAPP/PP2 prior probability of pathogenicity, which can be expected, since this parameter, which represents the starting point for Bayesian pathogenicity classification, is also strongly based on conservation assessment (**Table 3**).

For assessing the structural and functional roles of the residues, we generated an AlphaFold2 model of the MLH1-PMS2 heterodimer, which shows the structured N-terminal domains (NTD) containing the ATPases and the structured C-terminal domains (CTD) which harbor the endonuclease (**Figure 3B**). While the quality of these predictions is outstanding (1), we additionally relied on crystal structures of MutL protein fragments of human and other origin, since MutL proteins assume different conformations depending on whether ATP is bound or not (2, 3). During the repair process, the NTDs of MLH1 and PMS2 form a dimer when ATP is bound. As yet, the ensuing conformational rearrangements are only represented by one crystal structure, which has been resolved with the N-terminal dimer of *E.coli* MutL (3). Other available structures do not properly represent this since they only investigated isolated subunits. We assessed if the roles of the investigated residues are identical in the different structures and predictions used for the analysis; all investigated structures are listed in **Supplementary Table 4** (B1). The individual positions of all residues were identical in the investigated structures except for Asn335 (**Supplementary Table 4**, B1).

Asp286 is strongly conserved in eukaryotic PMS2 homologs (**Figure 3A**), but less conserved in other MutL proteins (MLH1 or bacterial MutL; **Table 2**) and therefore not a MutL family core functional residue. The residue is located in proximity of the N-terminal ATPase pocket of PMS2 (**Figure 3B**), but not involved in any direct contacts and oriented towards the exterior of the protein (**Supplementary Table 4**, B6). The crystal structure of human PMS2 (pdb 1h7s) confirms that it is located in a loop connecting two β-sheets of domain II of the PMS2-NTD at the solvent-exposed exterior of the MLH1-PMS2 heterodimer (**Figure 3C)** (4). In this loop, Arg282, Asp286 and Arg287 are three closely spaced, conserved residues in PMS2 (**Figure 3A and 3C**), where Asp286 can form a side-chain salt bridge with Arg282 (1h7s). The three charged residues likely provide a hydrophilic protein surface for this solvent-exposed loop. Such clusters of charged residues (both positive and negative) are typical for solvent-exposed protein surfaces (5). The loop is fixed in its position by several interactions, including a hydrogen bridge of the main chain carbonyl function of Asp286 with the side chain of Gln186 (blue in **Figure 3C**). However, the substitution of Asp286 to glycine therefore does not compromise the interaction with Gln186 since it is conferred by the main chain. The hydrophilic character of this area of the protein surface is also maintained by Arg282 and Arg287. Although a functional impact of the Asp286Gly substitution remains possible (**Supplementary Table 4**, *C*), it appears from our experimental data therefore that this alteration does not entail problems for the functionality of PMS2. This is consistent with the lack of functional or structural roles attributable to Asp286 and with the notion that substitutions of external residues are generally mostly neutral (6).

Asn335 is a residue absolutely conserved not only in PMS2 (**Figure 3A**), but throughout all MutL family proteins (**Table 2, Supplementary Table 4** A2/*A final*). This absolute conservation reflects its central role in the catalytic cycle of MutL proteins: it contributes to ATP-dependent, transient N-terminal dimerization (3) (**Supplementary Figure 4**). In different structures analyzed, it does not show identical orientation within the PMS2 protein (**Supplementary Table 4**, B1). For evaluating its role, it was therefore required to look not only at a structure of PMS2, but consider the conformational alterations that occur during formation of the transient ATP-dependent, N-terminal dimer of MLH1 and PMS2 that forms during the repair process. For this, there is currently only one representative structure available, which is the biological N-terminal dimer of *E.coli* MutL in the presence of a non-hydrolysable ATP analogue (PDB 1b63) (3). This structure demonstrates that the conserved Asn335 (Asn302 in *E.coli* MutL) forms a sidechain-sidechain interaction with a highly conserved arginine of the opposing subunit (Arg157 in *E.coli* MutL) and a side-chain-main-chain-interaction with Ile14 in the first helix of the opposing subunit (**Figure 3D**). A main chain interaction to a neighboring Asp300 of the same subunit stabilizes the conformation (**Figure 3D**). Not only Asn335, but all residues forming this N-terminal dimerization network (in PMS2: Asn335, Arg162, Asp333; see **Supplementary Figure 4B** for numbering) are absolutely conserved in MutL architecture, reflecting their pivotal roles in the MutL ATPase cycle. Together with another pair of side-chain salt-bridge interactions (Glu18-Arg95 in *E.coli*

MutL), these constitute a relevant force in N-terminal dimerization (**Supplementary Figure 4**). The alteration of Asn335 to serine will consequently cause a partial loss of the side-chain interactions to the opposing subunit. This will therefore affect N-terminal dimerization, and with it the ATPase cycle of MLH1-PMS2, which depends on N-terminal dimerization (3). It is therefore plausible that this alteration results in a functional deficit.

Ile679 at first sight seems not strongly conserved in PMS2 proteins (**Figure 3A**), but closer examination confirms that hydrophobic residues in this position are strongly conserved: only valine or leucine occur here besides isoleucine in PMS family proteins (**Figure 3A**, **Supplementary Table 4**, A3). Indeed, the hydrophobic nature of this position is conserved not only in PMS, but also in MLH1 family proteins (**Table 2**). This conservation finds an explanation in the structural position of the residue: PMS2 Ile679 contributes to the hydrophobic core of the MLH1-PMS2 dimer interface together with residues of MLH1 (Val531, Val534 and Leu540) between four β-sheets on each side (**Figure 3E, Supplementary Table 4** B2, B3 and B4). Introduction of the polar threonine residue in this position therefore will disrupt the hydrophobic interaction. It is therefore plausibly related to disturbing the dimerization of PMS2 with MLH1. Since the PMS2 protein is unstable in the absence of dimerization with MLH1, mutations disturbing interaction cause destabilization of PMS2 (7), which explains the reduced protein levels of PMS2 Ile679Thr (**Figure 1**). We have made a similar observation before with MLH1 Gln542Leu located at the rim of the MLH1-PMS2 dimerization interface (7). Both MLH1 Gln542Leu and PMS2 Ile679Thr appear to disrupt dimerization and thereby cause a defect in PMS2 protein levels and MMR activity. To our knowledge, this is the first report of a PMS2 missense variant disturbing MLH1-PMS2 dimerization. Consequently, the molecular role of this residue is fully consistent with the functional defect observed with Ile679Thr.

Arg799 is conserved in PMS2 homologues (**Figure 2A**) but not in other MutL proteins (**Table 2**), suggesting that this residue is probably not critical for MutL structure or architecture. It is located in a non-structured loop in the CTD (**Figure 3B**). Arg799 is directed into the protein's interior despite its charge, by means of its amphipathic nature (8). The nitrogen atoms of the guanidine group are positioned to make hydrogen bond contacts possible to three distant main chain carbonyl groups (Gln713, Tyr716 and Lys748). That way, Arg799 can contribute to tethering fractions of distantly located main chain, thus likely contributing to tertiary structure formation and stabilization. Arginine shares with tryptophane the amphipathic nature and the presence a nitrogen atom, which is located in identical distance to the main chain. Both arginine and tryptophan are able to use their lipophilic part for integration in a hydrophobic pocket while engaging in a hydrogen binding interaction using the nitrogen atoms (9, 10). Therefore, although tryptophane is bulkier and provides less nitrogen valences, it may replace arginine in certain occasions, compatible with the observation that Arg799Trp did not affect PMS2 stability and function.

1. Jumper J, Evans R, Pritzel A, Green T, Figurnov M, Ronneberger O et al. Highly accurate protein structure prediction with AlphaFold. Nature 2021.

2. Sacho EJ, Kadyrov FA, Modrich P, Kunkel TA, Erie DA. Direct visualization of asymmetric adenine-nucleotide-induced conformational changes in MutL alpha. Mol Cell 2008; 29(1):112–21.

3. Ban C, Junop M, Yang W. Transformation of MutL by ATP binding and hydrolysis: a switch in DNA mismatch repair. Cell 1999; 97(1):85–97.

4. Guarne A, Junop MS, Yang W. Structure and function of the N-terminal 40 kDa fragment of human PMS2: a monomeric GHL ATPase. EMBO J. 2001; 20(19):5521–31.

5. Zhu ZY, Karlin S. Clusters of charged residues in protein three-dimensional structures. Proc. Natl. Acad. Sci. U. S. A. 1996; 93(16):8350–5.

6. Iqbal S, Pérez-Palma E, Jespersen JB, May P, Hoksza D, Heyne HO et al. Comprehensive characterization of amino acid positions in protein structures reveals molecular effect of missense variants. Proc Natl Acad Sci U S A 2020; 117(45):28201–11.

7. Kosinski J, Hinrichsen I, Bujnicki JM, Friedhoff P, Plotz G. Identification of Lynch syndrome mutations in the MLH1-PMS2 interface that disturb dimerization and mismatch repair. Hum Mutat 2010; 31(8):975–82.

8. Betts MJ, Russell RB. Amino-Acid Properties and Consequences of Substitutions. In: Barnes MR, editor. Bioinformatics for geneticists: A bioinformatics primer for the analysis of genetic data. 2. ed. Chichester, Weinheim: Wiley; 2007. p. 311–42.

9. Flocco MM, Mowbray SL. Planar stacking interactions of arginine and aromatic side-chains in proteins. J Mol Biol 1994; 235(2):709–17.

10. Khemaissa S, Sagan S, Walrant A. Tryptophan, an Amino-Acid Endowed with Unique Properties and Its Many Roles in Membrane Proteins. Crystals 2021; 11(9):1032.

Supplementary Table 1: Primers used in RNA splicing analyses (minigene assays and analysis of patient's RNA)

Primer name[1]	Location	Primer sequence 5'-3' [2]	Purpose	
PMS2-ex8-NEBuilder-BamHI-F	*PMS2* intron 7	AAATAAGGCTAAGAAGTGCAGGATCCCATCAAGATTTGGAGCACAGATACC	Genomic DNA PCR amplification for minigene preparation	Minigene analysis
PMS2-ex8-recomb-R	*PMS2* intron 8	CAGCTAGGGGTCAAAACAAGACGCGTCAGTTGAAACATCTGAAGCTGGG		
PMS2-ex10-NEBuilder-BamHI-F	*PMS2* intron 9	AAATAAGGCTAAGAAGTGCAGGATCCTAGCACTTGTGATAAATACAAGTGAATGA		
PMS2-ex10-NEBuilder-MluI-R	*PMS2* intron 10	CAGCTAGGGGTCAAAACAAGACGCGTAAACTAGAGGTACTTGGAGGCAG		
PMS2-ex12-NEBrecomb-BamHI-F	*PMS2* intron 11	AAATAAGGCTAAGAAGTGCAGGATCCGGGTGACAGAACGAGACTCTGTTTC		
PMS2-ex12-NEBrecomb-MluI-R	*PMS2* intron 12	CAGCTAGGGGTCAAAACAAGACGCGTCTGTGTTGTCCGGGCTGGTC		
PMS2-ex14-NEBuilder-BamHI-F	*PMS2* intron 13	AAATAAGGCTAAGAAGTGCAGGATCCGTGAGTTTCCTCTCTTTCAGGTGT		
PMS2-ex14-NEBuilder-MluI-R	*PMS2* intron 14	CAGCTAGGGGTCAAAACAAGACGCGTCAAGGTCACAGAGAACGCAG		
PMS2-CL-c.75dup-F	*PMS2* intron 11	GTTTGACTTTTTTTTTATTACAGTGTTCTATAAC	Site-directed mutagenesis PCR to correct PMS2-CL variations (converting PMS2-CL sequence into PMS2) [3]	
PMS2-CL-c.61G>A-F	*PMS2* intron 12	CTAATAGAGGCCAAGGCAGGAGG		
PMS2-CL-c.61G>A-R	*PMS2* intron 12	CCTCCTGCCTTGGCCTCTATTAG		
PMS2-c.857A>G-F	*PMS2* exon 8	GGAGTTCAACAGGCAGACAGTTTTTC	Site-directed mutagenesis PCR to introduce the variants of interest[4]	
PMS2-c.857A>G-R	*PMS2* exon 8	GAAAAACTGTCTGCCTGTTGAACTCC		
PMS2-c.1004A>G-F	*PMS2* exon 10	GCGTTGATATCAGTGTTACTCCAGA		
PMS2-c.1004A>G-R	*PMS2* exon 10	TCTGGAGTAACACTGATATCAACGC		
PMS2-c.2036T>C-F	*PMS2* exon 12	GAAATGGAAATCACTGGTCAGTTTAACC		
PMS2-c.2036T>C-R	*PMS2* exon 12	GGTTAAACTGACCAGTGATTTCCATTTC		
PMS2-c.2395C>T-F	*PMS2* exon 14	GTCATGTGCTGGCCTTCC		
PMS2-c.2395C>T-R	*PMS2* exon 14	GGAAGGCCAGCACATGAC		
pCAS-seq.F	pCAS2 intron A	GGGGTCAATAGCAGTGAGAGG	Verification of minigene constructs by Sanger sequencing (sequencing of the full inserts and vector-insert junctions)	
pCAS-seq.R	pCAS2 intron B	GCTCCATTTCACAGGTAGAGA		
pCAS.KO1-F*	pCAS2 exon A	TGACGTCGCCGCCCATCAC	Analysis of minigene splicing patterns by RT-PCR and sequencing of gel-purified RT-PCR products	
pCAS-2.R	pCAS2 exon B	ATTGGTTGTTGAGTTGGTTGTC		
PMS2-RT-6F*	*PMS2* exon 6	AGCAGTTGCAAAGCCTCATT	Analysis of splicing patterns by RT-PCR and sequencing of gel-purified RT-PCR products	Patient's RNA analysis
PMS2-RT-11R2	*PMS2* exon 11	GCCTCTCGCAGTCTGGAAATG		
PMS2-ex10-F	*PMS2* intron 9	TAGCACTTGTGATAAATACAAGTGAATGA	Genomic DNA PCR amplification and sequencing	
PMS2-ex10-R	*PMS2* intron 10	AAACTAGAGGTACTTGGAGGCAG		
SNaPShot_c.1004A>G-R	*PMS2* exon 10	GATAGGGTTACAAAATTTGCCTTTTAT	Analysis of allele-specific expression by SNapShot	

[1] F, forward; R, reverse; *, fluorescent primer

[2] BamHI and MluI restriction sites are indicated in grey, and the position of the variant of interest is underlined

[3] The correction for *PMS2-CL* variations (PMS2 c.75del and PMS2 c.61A>G) detected in our initially obtained minigene pCAS2-PMS2-CL-"ex12"-WT was made in two subsequent steps:

Step 1 (correction of the first variant): First, and by using pCAS2-PMS2-CL-"ex12"-WT as template, we performed two parallel PCR reactions: one with the **PMS2-CL-c.75dup-F** forward primer and the **PMS2-ex12-NEBrecomb-MluI-R** reverse primer to yield PCR product **"A"**, and the second with **PMS2-ex12-NEBrecomb-BamHI-F** forward primer and the **PMS2-CL-c.61G>A-R** reverse primer to yield PCR product **"B"**. Then, these two PCR products ("A" and "B") were combined and used as template in a PCR reaction with primers **PMS2-ex12-NEBrecomb-BamHI-F** and **PMS2-ex12-NEBrecomb-MluI-R** to yield PCR product **"C"**. Finally, this PCR product ("C") was inserted into the initially obtained minigene pCAS2-PMS2-CL-"ex12"-WT *in lieu* of the corresponding unexpected PMS2-CL fragment to yield a partially corrected minigene

Step2 (correction of the second variant): First, and by using the partially corrected minigene as template, we performed a PCR reaction with the **PMS2-CL-c.61G>A-F** forward primer and the **PMS2-ex12-NEBrecomb-MluI-R** reverse primer to yield PCR product "**D**". Then, two PCR products ("**D**" and "**B**") were combined and used as template in a PCR reaction with primers **PMS2-ex12-NEBrecomb-BamHI-F** and **PMS2-ex12-NEBrecomb-MluI-R** to yield PCR product **"E"**. Finally, this PCR product ("E") was inserted into the partially corrected minigene *in lieu* of the corresponding unexpected PMS2-CL fragment to yield a corrected minigene entitled pCAS2-PMS2-ex12-WT

[4] The mutagenesis forward primers were paired with the reverse primer pCAS-seq-R, and the murtagenesis reverse primers were paired with the forward primer pCAS-seq-F

Supplementary Table 2: Primers for PMS2 protein experiments

Purpose	Variant name/protein description (NP_000526.2)	Variant cDNA description (NM_000535.7)	SNP identifier	Site-directed mutagenesis primer sequence F	Site-directed mutagenesis primer sequence R
Control	p.(Asp70Asn)	c.208G>A		TGAAGTTTCAaACAATGGATG	ATAAGATCCACTCCATAGTC
Variant #1	p.(Asp286Gly)	c.857A>G	rs116788608	AGTTCAACAGgCAGACAGTTTTTC	CCTTCCAACTCCATGCGT
Variant #2	p.(Asn335Ser)	c.1004A>G	rs200513014	GTTGATATCAgTGTTACTCCAG	GCATTCTGAATCAACAGAAATG
Variant #3	p.(Ile679Thr)	c. 2036T>C	rs778251286	gaaatcaCtggtcagtttaac	ctgaccaGtgatttccatttc
Variant #4	p.(Arg799Trp)	c.2395C>T	rs149202766	GGTCATGTGCtGGCCTTCCCG	CCAGGGCTGTCGCTCAGC

Supplementary Table 3A: Previously published analyses with the *in vitro* MMR system and comparison with InSiGHT classification

Gene	Variant	Work[1]	Functional analysis						Classification InSiGHT[2]	
			Expression % of WT	Reference		MMR activity % of WT	Functional result summary			
			>V716M=proficient <A681T=deficient	V716M	A681T	>75=proficient <25=deficient	Detail	in one word	Class	Class in words
MLH1	WT	1-10	100			100	Proficient (expression and MMR)			
	Asn38His	1	14	64	52	15	Deficient (expression and MMR)	deficient	5	pathogenic
	Asp41Gly	1	20	64	52	10	Deficient (expression and MMR)	deficient	5	pathogenic
	Asp41His	2	70	83	52	11	Deficient (MMR)	deficient	5	pathogenic
	Gly54Glu	1	52	64	52	44	unclear (both reduced)		-	no classification
	Gly65Asp	1	17	64	52	19	Deficient (expression and MMR)	deficient	4	likely pathogenic
	Gly67Arg	10	33	65	38	4	Deficient (expression and MMR)	deficient	5	pathogenic
	Leu73Pro	4	16	59	32	8	Deficient (expression and MMR)	deficient	3	uncertain
	Thr82Ala	10	66	65	38	3	Deficient (MMR)	deficient	4	likely pathogenic
	Thr82Ile	1	41	64	52	6	Deficient (expression and MMR)	deficient	5	pathogenic
	Lys84Glu	1	76	64	52	9	Deficient (MMR)	deficient	4	likely pathogenic
	Tyr97Asp	3	81	84	61	27	unclear (reduced MMR)		-	no classification
	Gly101Asp	1	16	64	52	11	Deficient (expression and MMR)	deficient	4	likely pathogenic
	Glu102Lys	1	2	64	52	27	Deficient (expression)	deficient	-	no classification
	His109Gln	1	71	64	52	71	unclear (reduced MMR)		3	uncertain
	His112Gln	3	103	84	61	72	unclear (reduced MMR)		-	no classification
	Thr117Met	1, 9	44	64	52	5	Deficient (expression and MMR)	deficient	5	pathogenic
	Ala128Pro	9	like wildtype			deficient	Deficient (MMR)	deficient	5	pathogenic
	Asp132His	9	like wildtype			like wildtype	Proficient (expression and MMR)	proficient	1	not pathogenic
	Pro141Ala	3	92	84	61	111	Proficient (expression and MMR)	proficient	3	uncertain
	Gly181Ser	5	110	70	37	115	Proficient (expression and MMR)	proficient	-	no classification
	Iso219Val	1, 10	94	64	52	97	Proficient (expression and MMR)	proficient	1	not pathogenic
	Arg265His	1	91	64	52	91	Proficient (expression and MMR)	proficient	3	uncertain
	Arg265Pro	3	60	84	61	29	Deficient (expression)	deficient	4	likely pathogenic
	Asn338Ser	3,5	130	84	61	95	Proficient (expression and MMR)	proficient	3	uncertain
	Tyr379Cys	6	110	n.t.	n.t.	94	Proficient (expression and MMR)	proficient	2	likely not pathogenic
	Val384Asp	6	112	n.t.	n.t.	92	Proficient (expression and MMR)	proficient	1	not pathogenic
	Arg385Pro	6	116	n.t.	n.t.	13	Deficient (MMR)	deficient	3	uncertain
	Arg385Cys	6	105	n.t.	n.t.	87	Proficient (expression and MMR)	proficient	-	no classification
	Arg385His	6	86	n.t.	n.t.	100	Proficient (expression and MMR)	proficient	-	no classification
	Ile500del	3	105	84	61	104	Proficient (expression and MMR)	proficient	-	no classification
	Val506Ala	1	58	64	52	88	unclear (reduced expression)		3	uncertain
	Leu507Phe	2	101	83	52	91	Proficient (expression and MMR)	proficient	-	no classification
	Arg522Trp	1	62	64	52	105	unclear (reduced expression)		-	no classification
	Gln542Leu	7	109	n.t.	n.t.	reduced	unclear (reduced MMR)		3	uncertain
	Asn551Thr	1	53	64	52	80	unclear (reduced expression)		3	uncertain
	Leu559Arg	1	45	64	52	79	Deficient (expression)	deficient	3	uncertain
	Leu574Pro	1	27	64	52	16	Deficient (expression and MMR)	deficient	4	likely pathogenic
	Arg575Lys	3	121	84	61	101	Proficient (expression and MMR)	proficient	-	no classification
	Glu578Gly	1	71	64	52	91	Proficient (expression and MMR)	proficient	1	not pathogenic
	Ala586Pro	1	50	64	52	21	Deficient (expression and MMR)	deficient	3	uncertain
	Asp601Gly	1	82	64	52	96	Proficient (expression and MMR)	proficient	3	uncertain
	Glu605del	2	70	83	52	89	unclear (reduced expression)		-	no classification
	Glu605del+Val716Met	2	44	83	52	87	Deficient (expression)	deficient	-	no classification
	Lys616del	3	40	84	61	35	Deficient (expression)	deficient	-	no classification
	Lys618Ala	1	78	64	52	92	Proficient (expression and MMR)	proficient	1	not pathogenic
	Leu622His	1, 10	42	64	52	80	Deficient (expression)	deficient	5	pathogenic
	Leu636Pro	7	40	n.t.	n.t.	proficient	unclear (reduced expression)		3	uncertain
	Pro640Ser	1	30	64	52	80	Deficient (expression)	deficient	4	likely pathogenic
	Pro640Thr	4	31	59	32	66	Deficient (expression)	deficient	-	no classification
	Pro648Leu	1	22	64	52	92	Deficient (expression)	deficient	4	likely pathogenic
	Leu653Arg	1	11	64	52	5	Deficient (expression and MMR)	deficient	4	likely pathogenic
	Pro654Leu	1	25	64	52	67	Deficient (expression)	deficient	5	pathogenic
	Arg659Gln	1	61	64	52	85	unclear (reduced expression)		3	uncertain
	Arg659Leu	1	28	64	52	8	Deficient (expression and MMR)	deficient	-	no classification
	Arg659Pro	1	13	64	52	99	Deficient (expression)	deficient	5	pathogenic
	Thr662Pro	1	22	64	52	93	Deficient (expression)	deficient	-	no classification
	Glu663Gly	1	85	64	52	95	Proficient (expression and MMR)	proficient	-	no classification
	Leu676Arg	1	24	64	52	12	Deficient (expression and MMR)	deficient	3	uncertain
	Leu676Pro	3	41	84	61	49	Deficient (expression)	deficient	3	uncertain
	Gln689Arg	2	106	83	52	99	Proficient (expression and MMR)	proficient	1	not pathogenic
	His718Tyr	1	73	64	52	110	Proficient (expression and MMR)	proficient	1	not pathogenic
	Arg725Cys	1	26	64	52	101	Deficient (expression)	deficient	3	uncertain
	Glu736Lys	4	83	59	32	83	Proficient (expression and MMR)	proficient	-	no classification
	Leu749Pro	1	85	64	52	15	Deficient (MMR)	deficient	5	pathogenic
	Tyr750X	7	126	n.t.	n.t.	reduced	unclear (reduced MMR)		3	uncertain
	Arg755Trp	1, 7	72	64	52	10	Deficient (MMR)	deficient	3	uncertain
	Arg755Ser	7	111	n.t.	n.t.	n.t.				
PMS2	Arg20Gln	8	82	n.a.	n.a.	89	Proficient (expression and MMR)	proficient	1	not pathogenic
	Asp70Gln	8	90	n.a.	n.a.	8	Deficient (MMR)	deficient	-	no classification
	Ser46Ile	8	108	n.a.	n.a.	14	Deficient (MMR)	deficient	4	likely pathogenic
	Pro470Ser	8	98	n.a.	n.a.	90	Proficient (expression and MMR)	proficient	1	not pathogenic

Number of variants with functional testing result (total number in list):	71
Number of variants with classifications, either "(likely) pathogenic" or "(likely) not pathogenic" (Classes 1,2, 4 and 5):	31
**Number of variants classified "pathogenic" or "likely pathogenic" which were tested deficient (consistent results):**	**21**
**Number of variants classified "not pathogenic" or "likely not pathogenic" which were tested proficient (consistent results):**	**10**

[1] Work (Literature; for full reference information, see main manuscript reference list):

1. Hinrichsen *et al.* 2013
2. Hinrichsen *et al*. 2014
3. Köger *et al*. 2018
4. Mahdouani *et al*. 2022
5. Mahdouani *et al*. 2024
6. Wolf *et al*. 2023
7. Kosinski *et al.* 2010
8. Borras *et al*. 2013
9. Plotz *et al.* 2006
10. Gonzalez-Acosta *et al.* 2019

[2] Database interrogated in January 2025 (https://www.insight-database.org/classifications/)

Supplementary Table 3B: Odds of Pathogenicity (OddsPath) estimated by performance of classified variant controls

According to Table S1 in Additional file 1 of Brnich SE et al., Genome Medicine 12 (2019): https://doi.org/10.1186/s13073-019-0690-2

Note: this estimate "adds 1.0" to the numerator and denominator in order to do a calculation

	# Classified			Proportion pathogenic (Prior, P_1)	Assay result		"+1 proportions" (Posterior, P_2)		OddsPath=$[P_2 \times (1-P_1)] / [(1-P_2) \times P_1]$	
	Total	Pathogenic	Benign		Functionally abnormal	Functionally normal	Pathogenic	Benign	Pathogenic	Benign
MLH1 and PMS2 variants	31	21	10	0,68	21	10	0,95	0,09	8,9	0,048
MLH1 variants	28	20	8	0,71	20	8	0,95	0,11	7,8	0,050
PMS2 variants	3	1	2	0,33	1	2	0,50	0,33	1,5	1,000

Odds of Pathogenicity (OddsPath)	Evidence strength equivalent
<0.0029	BS3_very_strong^
<0.053	BS3
<0.23	BS3_moderate*
<0.48	BS3_supporting
0.48-2.1	Indeterminate
>2.1	PS3_supporting
>4.3	PS3_moderate
>18.7	PS3
>350	PS3_very_strong

^There are no very strong benign evidence codes included in the Richards, et al. guidance. This category is included here for modeling

*Since there are no moderate strength benign evidence codes included in the Richards, et al. guidance, moderate level evidence is equivalent to two instances of supporting-level benign evidence

Supplementary Table 4: MMR Protein Residue Conservation and Structure Assessment Form (*ConStruct Assessment*)*

A	CONSERVATION[1]	Asp286Gly	Asn335Ser	Ile679Thr	Arg799Trp
A 1	*PMS2 family alignment evaluated number of sequences of different organisms*	926			
	MLH1 family alignment evaluated number of sequences of different organisms	953			
	MutL family alignment evakuated number of sequences of different organisms	96			
	Corresponding residue in MLH1 (referring to sequence Homo sapiens MLH1 Uniprot P40692)	*Iso257*	*Asn306*	*Val531*	*Asn665*
	Corresponding residue in MutL from bacteria (referring to sequence Bacillus subtilis MutL Uniprot P49850)	*Asn247*	*Asn297*	*Iso442*	*Iso560*
A 2	Conservation in PMS2 family of proteins	9	9	8[10]	9
	Conservation of the corresponding residue in MLH1 protein family	6	9	9	7
	Conservation of the corresponding residue in bacterial MutL family (without gammaproteobacteria expressing MutH)	7	9	7[10]	5
A 3	Hydrophobic residue (conservation may include alternative hydrophobic residues)	N	N	Y	N
	Y Are hydrophobic residues conserved in this position (which)?			Y (I,V,L)	
A final	Is the residue conserved in its subfamily (Y; Y(h=conservation of hydrophobicity)[8]; N)?	Y	Y	Y(h)	Y
	Is the residue conserved in the complete MutL family (Y; Y(h=conservation of hydrophobicity)[8]; N)	N	Y	Y(h)	N
**B**	**STRUCTURE**	**Asp286Gly**	**Asn338Ser**	**Ile679Thr**	**Arg799Trp**
B 1	*Structural analyses performed with: PDB identifier(s), corresponding residue if structure is from another organism:*	*AlphaFold2 Model*[2]	*AlphaFold2 Model*[2]	*AlphaFold2 Model*[2]	*AlphaFold2 Model*[2]
		1H7S	*1H7S*	*4E4W (S.cerevisiae: Val677)*	*4E4W (S.cerevisiae: Arg804)*
			1B63 (E.coli: Asn302)		
	Is the relative position/role of the side chain identical in all investigated structures?	*Yes*	*No*[9]	*Yes*	*Yes*
B 2	**Functional or sensitive regions (with potentially increased likelihood of detrimental effects of substitutions)**	**Asp286Gly**	**Asn338Ser**	**Iso679Thr**	**Arg799Trp**
	Located in the ATP binding domain?		Y		
	Located in the N-terminal dimerization interface?		Y		
	Located in the C-terminal dimerization interface?			Y	
	Located in the endonuclease active site?				
	MLH1: Located in the structurally sensitive CTD region[3] ?				
	MLH1: Located in the conserved linker region?				
	PMS2: Located in the conserved linker region?				
B 3	**Secondary structure** [4]	**Asp286Gly**	**Asn338Ser**	**Ile679Thr**	**Arg799Trp**
	Is the residue located within a secondary structure (which)?	N (loop)	N (loop)	Y (β-sheet)	N (loop)
	Y May the exchange interfere with formation of the secondary structure (e.g. side chain involved in secondary structure stabilization)? Use of Jpred suggested.			Unlikely (Jpred4)	
B 4	**Side-chain hydrophobic interactions – protein interior** [5]	**Asp286Gly**	**Asn338Ser**	**Ile679Thr**	**Arg799Trp**
	Is the residue oriented towards the interieur of the protein (where)?	N	N	Y (CT-dimer interface)	Y (CTD)
	Y Is the residue engaged in a hydrophobic internal interaction (other hydrophobic side chains present to form a hydrophobic core)?			Y	N
	Y Are the other hydrophobic side chains conserved (in hydrophobicity)? Which?			Y (V531, V534, L540)	
	Y May the exchange interfere with the hydrophobic interaction based on chemistry?			**Y (exchange for a bulky polar residue in the hydrophobic C-terminal dimerization interface)**	
B 5	**Side-chain polar interactions – protein interior** [6]	**Asp286Gly**	**Asn338Ser**	**Ile679Thr**	**Arg799Trp**
	Y Is the side-chain engaged in a salt bridge or polar interaction?	N	Y	N	Y
	Y Is the interaction partner another side-chain (which)?		Y (MLH1 Arg162)		
	Y Is the other side-chain conserved?		Y		
	Y May the exchange disturb the interaction?		**Y (NT-dimer interation)**		
	Y Is the interaction partner a main chain atom?		Y		Y
	Y Is the interaction partner a distant atom, so possibly relevant for tertiary structure formation?		Y (MLH1 Iso14)		Y (Q713-T716-K748)
	Y May the exchange disturb the interaction?		**Y (NT-dimer interation)**		unclear
B 6	**Side-chain surface interactions/protein exterieur** [7]	**Asp286Gly**	**Asn338Ser**	**Ile679Thr**	**Arg799Trp**
	Is the residue located on the surface of the protein (exposed)?	Y	N	N	N
	Y Is the residue located within a known protein-protein interaction site?	N			
	Y Is the residue located within a conserved protein surface?	N[11]			
	Is the side-chain engaged in a salt bridge or polar interaction?	Y			
	Y Is the interaction with another side-chain (which)?	Y (R282 and R287)			
	Y Is the other side-chain conserved?	Y			
	Y May the exchange disturb the interaction?	Y			
	Y Is the interaction with a main chain atom?	Y			
	Y May the exchange disturb the interaction?	Y			
	Y Is it a distant interaction (not within the same ss, so possibly relevant for tertiary structure formation?	N			
**C**	**FUNCTIONAL IMPACT considered unlikely/possible/likely? (Reason)**	**Possible (B6)**	**Likely (A final, B5)**	**Likely (A final, B4)**	**Possible (B5)**

* The procedure is explained in more detail in Supplementary Material.

[1] Assessed as ConSeq score using as detailed in Materials and Methods. The conservation in other protein family groups (MLH1, MutL), represents the score of the corresponding position in their respective alignment.

[2] Model: AlphaFold2 model of the complete MLH1-PMS2 heterodimer (see Materials and Methods)

[3] See publication: Hinrichsen *et al*., Clin Canc Res 2013 May 1;19(9):2432-41

[4] Substitutions in β-sheets are frequently detrimental, while location in unstructured region, coils or helix is frequently neutral. See publication: Iqbal *et al*., PNAS 2020 Nov 10;117(45):28201-28211

[5] Substitutions of internal residues are frequently detrimental. See publication: Iqbal *et al*., PNAS 2020 Nov 10;117(45):28201-28211

[6] Substitutions of internal residues are frequently detrimental. See publication: Iqbal *et al*., PNAS 2020 Nov 10;117(45):28201-28211

[7] Substitutions of external residues are frequently neutral. See publication: Iqbal *et al*., PNAS 2020 Nov 10;117(45):28201-28211

[8] Hydrophobic interactions may be conserved in conservative substitutions which retain the hydrophobic character of the residue.

[9] As a consequence, the residue's role was studied in the bacterial structure which represents the biologically relevant N-terminal dimer (1B63)

[10] Lower degree of conservation results from conservation of different hydrophobic residues; therefore, hydrophobicity is highly conserved in this position.

[11] See Supplementary Figure 5
